# *Lactobacillus* spp. in the reproductive system of female moths and mating induced changes and possible transmission

**DOI:** 10.1186/s12866-022-02724-6

**Published:** 2022-12-19

**Authors:** Qing-Yi Zhao, Luo-Yan Zhang, Da-Ying Fu, Jin Xu, Peng Chen, Hui Ye

**Affiliations:** 1 Yunnan Academy of Biodiversity, Southwest Forestry University, Bailong Road 300#, Kunming, 650224 China; 2 Key Laboratory for Forest Resources Conservation and Utilization in the Southwest Mountains of China, Ministry of Education, Southwest Forestry University, Bailong Road 300#, Kunming, 650224 China; 3 Yunnan Academy of Forestry and Grassland, Lanan Road 2#, Kunming, 650201 China; 4 School of Ecology and Environment, Yunnan University, Cuihu North Road 2#, Kunming, 650091 China

**Keywords:** *Spodoptera frugiperda*, *Lactobacillus*, Reproductive system, Gut, Mating, Sexually transmitted microbes, Healthy vaginal microbiome

## Abstract

**Background:**

The microbiome in the insect reproductive tract is poorly understood. Our previous study demonstrated the presence of *Lactobacillus* spp. in female moths, but their distribution and function remain unclear. *Lactobacillus* spp. are known as the ‘healthy’ vaginal microbiome in humans.

**Results:**

Here, we studied the microbiome in the reproductive system (RS) and gut of *Spodoptera frugiperda* using 16S rDNA sequences. The obtained 4315 bacterial OTUs were classified into 61 phyla and 642 genera, with Proteobacteria, Firmicutes and Bacteroidota being the top three dominant phyla and *Enterococcus* and *Asaia* being dominant genera in most samples. Mating dramatically increased the abundance of pathogens or pathogenic functions in the gut, while in the RS, the change range was trivial. Taxonomy assignment identified thirteen *Lactobacillus* spp. in *S. frugiperda*, with *Lactobacillus crustorum* and *Lactobacillus murinus* showing high abundance. Three species found in *S. frugiperda*, namely *L. reuteri*, *L. plantarum* and *L. brevis*, have also been identified as human ‘healthy’ vaginal bacterial species. *Lactobacillus* spp. showed higher abundance in the RS of virgin females and lower abundance in the RS of virgin males and the gut of virgin females. Mating reduced their abundance in the RS of females but increased their abundance in the RS of males, especially in males mated with multiple females. The RS of virgin females and of multiple mated males were very similar in terms of composition and abundance of *Lactobacillus* species, with *Lactobacillus crustorum* showing much higher abundance in both tissues, potentially due to sexual transmission.

**Conclusions:**

*Lactobacillus* spp. showed high abundance and diversity in the RS of female moths. The higher abundance of *Lactobacillus* spp. in the RS of female moths and the similarity of *Lactobacillus* species in female moths with human ‘healthy’ vaginal *Lactobacillus* spp. suggest that these bacterial strains are also an important microbiome in the RS of female moths.

**Supplementary Information:**

The online version contains supplementary material available at 10.1186/s12866-022-02724-6.

## Background

To date, a large number of studies have been conducted to reveal the role of the gut microbiome in different animals and have provided deep insight from the perspectives of ecology, adaptation and evolution [1–5]. These studies have shown that gut microbes may contribute to the host by providing nutritional components, promoting digestive efficiency, and helping the host to live on a suboptimal environmental diet. For example, nitrogen fixation by gut microbiota in some termites can account for up to 60% of the nitrogen obtained by the host [6]. In the human gut, bacterial fermentation products, such as short-chain fatty acids, have been shown to play anti-inflammatory and protective roles [3]. Resident gut bacteria may also benefit insects by providing protection against colonization of the gut by pathogens [7] or by counteracting plant toxic defences [8].

Studies in mammals have revealed a diversity of microbial residents in reproductive organs and sexually transmitted microbes [9, 10]. Some of these microbes may have significant effects on the survival and reproductive success of males and females [9, 10]. Studies in male mammals have recognized that some of the sexually transmitted microbes have negative effects on sperm quality and fertility [9–11]. Evidence from female mammals increasingly recognizes that some of these microbes are associated with female reproduction, such as infertility, preterm birth and sexually transmitted diseases [10, 12–15]. Moreover, studies in humans have also identified some ‘healthy’ vaginal microbes, such as *Lactobacillus* spp., which are typically the dominant vaginal microbes. These ‘healthy’ microbes are oxygen-tolerant anaerobes and exhibit antimicrobial activity against a range of vaginal pathogens, probably by producing of lactic acid, stimulating host defence responses and physically barring against pathogen adhesion [13, 14, 16, 17]. However, the microbiota in insect reproductive organs is relatively less well known [9, 18]. Evidence for such reproduction-related pathogens or healthy microbes is especially limited in insects. A study by Otti et al. [19] showed that exposing bedbugs to polymicrobial mixtures (including *Bacillus*, *Staphylococcus*, *Acinetobacter* and *Alcaligenes*) resulted in high sperm mortality (up to 40%). Studies in fruit flies have also demonstrated that some *Enterococcus* species*,* such as *E. faecalis,* may have a negative impact on the fecundity of the host [20, 21]. However, no such ‘healthy’ vaginal microbes have been identified in insects thus far.

Furthermore, studies in humans have demonstrated that the microbiome of the female gut and reproductive organs represent very complex biological ecosystems; they communicate with each other and cause widespread impacts on the host, such as functions in host immunological and metabolic homeostasis [3]. These vaginal microbes may derive from the gut by continuous translocation and have evolved to adapt to the vaginal microenvironment [22, 23]. However, the functions elicited by these microbes and their metabolic byproducts within reproductive organs seem to be different from those in the gut [3]. For example, bacterial short-chain fatty acids play anti-inflammatory and protective roles in the gut, while they may exhibit dysbiotic and proinflammatory effects in the genital tract [3, 24]. Therefore, study of the gut and genital tract microbiome-induced crosstalk is helpful to achieve new findings in this field.

One possible reason for the limited evidence on such a reproduction-related microbiome in insects is that most of these microbes are uncultivable [18]. Recent developments in high-throughput sequencing and bioinformatics have provided an effective approach for understanding the symbiotic microbiome. Studies in a few insects have revealed the bacterial communities in their reproductive organs by using 16S rDNA sequencing and bioinformatic analysis [18, 25–27]. Sequencing and analysis in the Chinese citrus fly *Bactrocera minax* revealed that the female ovary has a higher diversity of the microbiome than the male testis, and the bacterial diversity of reproductive organs is higher than that of the gut [26]. Bellinvia et al. [18, 25] recently reported that the genital microbiome is sexually transmittable between sexes and may play roles in shaping the evolution of reproductive traits [18, 25]. However, no studies have tested the effect of mating on the microbiota both in the gut and reproductive organs.

Mating may have a significant (negative or positive) effect on female immunity [28, 29], which may also affect the abundance and diversity of the reproduction-related microbiome. Mating may negatively affect female immune activity due to trade-offs between reproduction and survival [28] and males may directly suppress female immunity to promote sperm storage and egg fertilization [29]. Studies have also indicated that mating may upregulate the female immune response due to the transfer of foreign materials and mating induced infections [30–33]. Females of polygamous species thus may have higher post-mating immunity if sexually transmitted infection is the major factor driving female postmating costs [31]. Sexually transmitted infection may negatively affect female lifespan and reproductive output in insects [34]. Therefore, it will be interesting and informative to study the reproductive microbiome in different mating systems under different mating conditions.

The fall armyworm (*Spodoptera frugiperda*) is currently a major worldwide agricultural pest, that mainly attacks corn, rice, wheat, sorghum and cotton [35]. This moth pest is native to tropical and subtropical regions in the Americas [36]. It was first discovered in the southwestern China at the end of 2018, and then spread to vast areas of China soon after [37]. This pest is notorious due to its long-distance migration ability [38], high fecundity [39] and strong pesticide resistance [40, 41]. Environmentally friendly and sustainable management strategies, such as modifying microbial communities [42–44], are required for better control of this pest in the future.

Our previous study in *S. frugiperda* using the whole abdomen of female adults provided interesting results, wherein mating caused a decline in the diversity of symbiotic microbiomes and promiscuity incurred a higher pathogen abundance [45]. In the present study, we further studied the diversity and abundance of the microbiome in the reproductive system (both male and female) and gut (female) of *S. frugiperda*, and mating caused changes and transmissions. The evolutionary significance and crosstalk between the gut and reproductive organ microbiomes and between the host and microbiome are explored and discussed.

## Materials and methods

### Insect rearing and sampling

The larvae of *S. frugiperda* were collected in corn fields near Dongchuan town in Yunnan Province, China. The larvae were reared on an artificial diet [46] under 28 ± 1 °C and 60–80% relative humidity with a 14:10-h light:dark photoperiod. Adults were fed a 10% honey solution. The offspring were used in the present study.

**Fig. 1:**
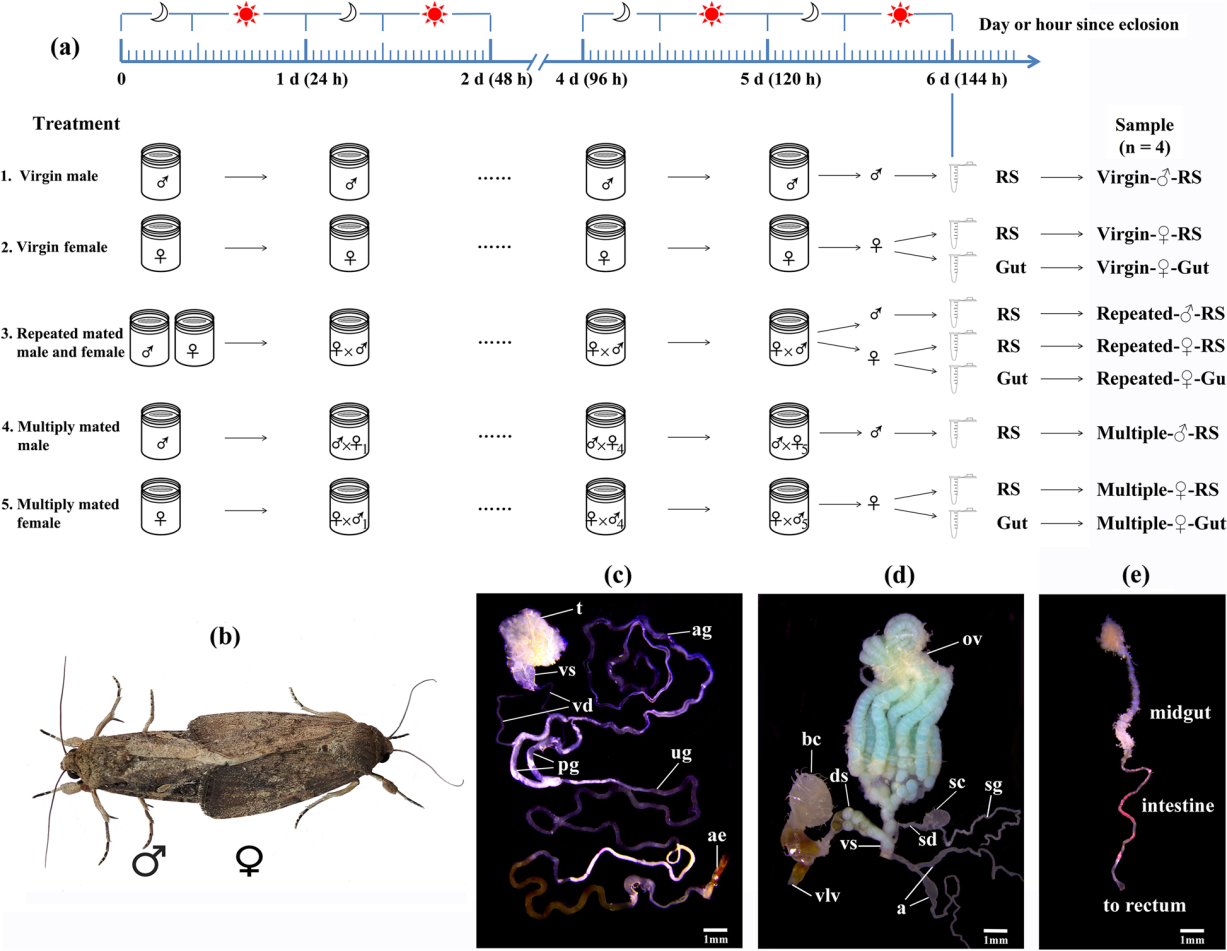
Experimental design and sample information of *S. frugiperda*. **a** Treatment and sampling method and sample name; **b** A male and a female mating in *S. frugiperda*; **c** Sampled male reproductive system, in which ae = Aedeagus, ag = Accessory gland, pg = Paired gland, t = Testis, ug = Unpaired gland, vd = Vas deferens, and vs = Vesicula seminalis; **d** Sampled female reproductive system, in which a = Accessory gland reservoir, bc = Bursa copulatrix, ds = Ductus seminalis, ov = Ovary, sc = Spermatheca, sd = Spermathecal duct, sg = Spermathecal gland, vlv = Vulva, and vs = Vestibulum; and **e** Sampled female gut

To ensure virginity and age, male and female pupae were sexed based on morphological characteristics [47] and then caged separately. Newly eclosed adults were collected and used for the following treatment and sampling. To sample the male reproductive system (RS) and female RS and gut under different mating conditions, five treatments were established (Fig. 1a): (1) virgin males were individually caged from the day of eclosion until sampling, and their RS were sampled to obtain the sample of Virgin-♂-RS; (2) virgin females were individually caged from the day of eclosion until sampling, and their RS and gut were sampled to obtain Virgin-♀-RS and Virgin-♀-Gut, respectively; (3) virgin males and females were paired permanently with one pair per box from the second day since eclosion until sampling, and males and females mated at least two times with the same mates were used for RS and gut sampling to obtain the Repeated-♂-RS, Repeated-♀-RS and Repeated-♀-Gut; (4) a virgin male was provided with one novel age-matched virgin female each night from the second day since eclosion until sampling, and males mated at least two times with different females were used for RS sampling to obtain Multiple-♂-RS; and (5) a virgin female was provided with one novel age-matched virgin male each night from the second day since eclosion until sampling, and females mated at least two times with different males were used for RS and gut sampling to obtain Multiple-♀-RS and Multiple-♀-Gut. Males and females were paired during the night but separated during the daytime. The mating events (Fig. 1b) of all pairs were recorded daily by quickly observing treated insects every 30 min (the mating duration was approximately 1 h [48]). The experimental conditions were the same as described above. Each box was supplied with a 10% honey solution as food. A 15 W red light was used for illumination during observation. After the mating treatments, the male RS and female RS and gut were sampled at the end of the 6th day after eclosion through dissection. The males and females that were mated repeatedlly mated two to five times before sampling had mating times of 2.72 ± 0.07 (mean ± SE). The multiply mated males or females, which were also mated two to five times, had mating times of 3.02 ± 0.06 and 2.83 ± 0.06, respectively. Whole bodies of males or females were rinsed twice with sterile water, surface-sterilized in 75% ethanol for 90 s, and then rinsed twice again using sterile water. The moths were dissected to obtain the male RS (Fig. 1c) or female RS (Fig. 1d) and gut (Fig. 1e) in a plate containing 10 ml sterile PBS buffer (pH 7.4) under a stereomicroscope. To obtain sufficient tissue for sequencing, 30 RS or gut (from 30 moths) were combined to form a sample replicate. Four biological replicates were used for each sample (*n* = 4). The samples were stored at − 80 °C until use.

### DNA extraction and 16S rDNA sequencing

The CTAB method [49] was used to extract total genomic DNA from samples, and 1.0% agarose gel electrophoresis was used to examine the DNA purity and concentration. The 16S rDNA regions were amplified via PCR using Phusion High-Fidelity PCR Master Mix (New England Biolabs, USA) and the V3-V4 primers (515F: 5′-GTGYCAGCMGCCGCGGTAA-3′; 806R: 5′-GGACTACNNGGGTATCTAAT-3′). The PCR products were analysed using 2% agarose gel electrophoresis and purified with a GeneJET Gel Extraction Kit (Thermo Scientific, USA). Sequencing libraries were prepared using a TruSeq DNA PCR-Free Library Preparation Kit (Illumina, USA) following the manufacturer’s protocol. The library quality was assessed using a Qubit Fluorometer (Thermo Scientific, USA) and an Agilent Bioanalyzer 2100 system (Agilent Technologies, USA). Finally, the library was submitted for 16S rDNA sequencing using the Illumina NovaSeq PE250 platform at Beijin Novogene Bioinformatics Technology Co., Ltd.. The obtained raw data were deposited into the NCBI SRA database (Project No.: PRJNA844607).

### Data analysis

Raw sequencing reads were assembled to obtain raw tags using FLASH software (v1.2.7, http://ccb.jhu.edu/software/FLASH/) [50]. Subsequently, clean tags were obtained after filtering low-quality and short-length raw tags with fastp v0.23.0 [51]. The effective tags were obtained by filtering the chimeric sequences in the raw tags using VSEARCH software (v2.19.0, https://github.com/torognes/vsearch/) [52]. The values of Q20 and Q30 of the effective tags were calculated to evaluate the sequence quality. Effective tags were analysed using UPARSE software (UPARSE v7.0.1001, http://drive5.com/uparse/) [53]. Sequences with ≥97% similarity were assigned to the same OTUs. A representative sequence for each OTU was screened for subsequent annotation by the Silva Database (https://www.arb-silva.de/) [54] based on the Mothur algorithm.

Alpha diversity was performed to reveal the complexity of communities within samples using QIIME (Version 1.7.0). Alpha diversity indices, including Observed species, Shannon, Simpson, Chao1 and Good’s coverage indices, were calculated. The differences in Shannon indices between samples were further analysed using ANOVA followed by LSD tests with Benjamini–Hochberg correction for multiple comparisons. Beta diversity was applied to assess the differences in the microbial community between samples. The significance of differences among or between samples was tested by permutational multivariate analysis of variance (PERMANOVA) based on Bray–Curtis metrics and then visualized accordingly by principal coordinates analysis (PCoA). PERMANOVA was performed using R (Version 4.0.3) with the vegan and phyloseq packages.

Linear discriminant analysis of effect size (LEfSe) (http://huttenhower.sph.harvard.edu/galaxy/) was used to determine OTUs that discriminate among the populations with an LDA score greater than 3.0.

Functional annotation of prokaryotic taxa (FAPROTAX) [55] was used to predict the potential functional annotation of bacterial taxa in different samples, which predicts functions of uncultured prokaryotes from the known functions of cultured bacterial genera. FAPROTAX is a promising tool for predicting ecologically relevant functions of bacterial and archaeal taxa derived from 16S rDNA amplicon sequencing [56].

## Results

### Sequencing and quality control

By 16S rDNA sequencing, ~ 82,300 effective tags were obtained from each of the 36 libraries, with an average length of 417–428 bp (Table S1). The percentages of Q20 and Q30 of all samples’ effective tags were 98.42–99.24% and 94.74–96.95%, respectively, indicating that the assembly quality was good. These effective tags were clustered into 4315 OTUs (Table S2). Rarefaction analysis showed a saturating number of OTUs (Fig. S1), indicating an adequate sequencing output for all samples.

### Diversity indices of bacterial OTUs

The Good’s coverages were all greater than 99% for all samples (Table 1), suggesting that the number of clones sampled was sufficient to provide an adequate estimation of bacterial diversity in *S. frugiperda*. The number of OTUs in different samples ranged from 1047 to 2091 (Table 1). Approximately 5% (216/4315%) of OTUs were shared by all samples (Fig. S2).

**Table 1:** Alpha diversity indices of bacteria in different samples of *S. frugiperda*

Sample name	Number of OTUs	Observed species	Shannon	Simpson	Chao1	Cood’s coverage
Virgin-♂-RS	1188	488 ± 87	3.760 ± 0.151	0.8542 ± 0.0187	564.85 ± 105.72	0.9978 ± 0.00048
Repeated-♂-RS	2091	925 ± 98	4.366 ± 0.246	0.8813 ± 0.0123	1038.29 ± 100.42	0.9965 ± 0.00029
Multiple-♂-RS	1047	409 ± 90	3.752 ± 0.348	0.8525 ± 0.0290	465.50 ± 106.31	0.999 ± 0.00050
Virgin-♀-RS	2057	753 ± 115	4.505 ± 0.132	0.8505 ± 0.0213	818.33 ± 120.07	0.997 ± 0.00065
Repeated-♀-RS	1094	380 ± 117	3.101 ± 0.172	0.7742 ± 0.0271	446.22 ± 129.61	0.998 ± 0.00048
Multiple-♀-RS	1597	531 ± 139	4.099 ± 0.514	0.8622 ± 0.0419	596.30 ± 158.78	0.998 ± 0.00063
Virgin-♀-Gut	1215	564 ± 65	3.512 ± 0.357	0.7708 ± 0.0419	628.05 ± 70.90	0.998 ± 0
Repeated-♀-Gut	2041	845 ± 125	4.649 ± 0.297	0.8740 ± 0.0195	937.89 ± 150.30	0.997 ± 0.00095
Multiple-♀-Gut	1283	471 ± 79	3.442 ± 0.200	0.8020 ± 0.0394	544.62 ± 92.29	0.998 ± 0.00041

**Fig. 2:**
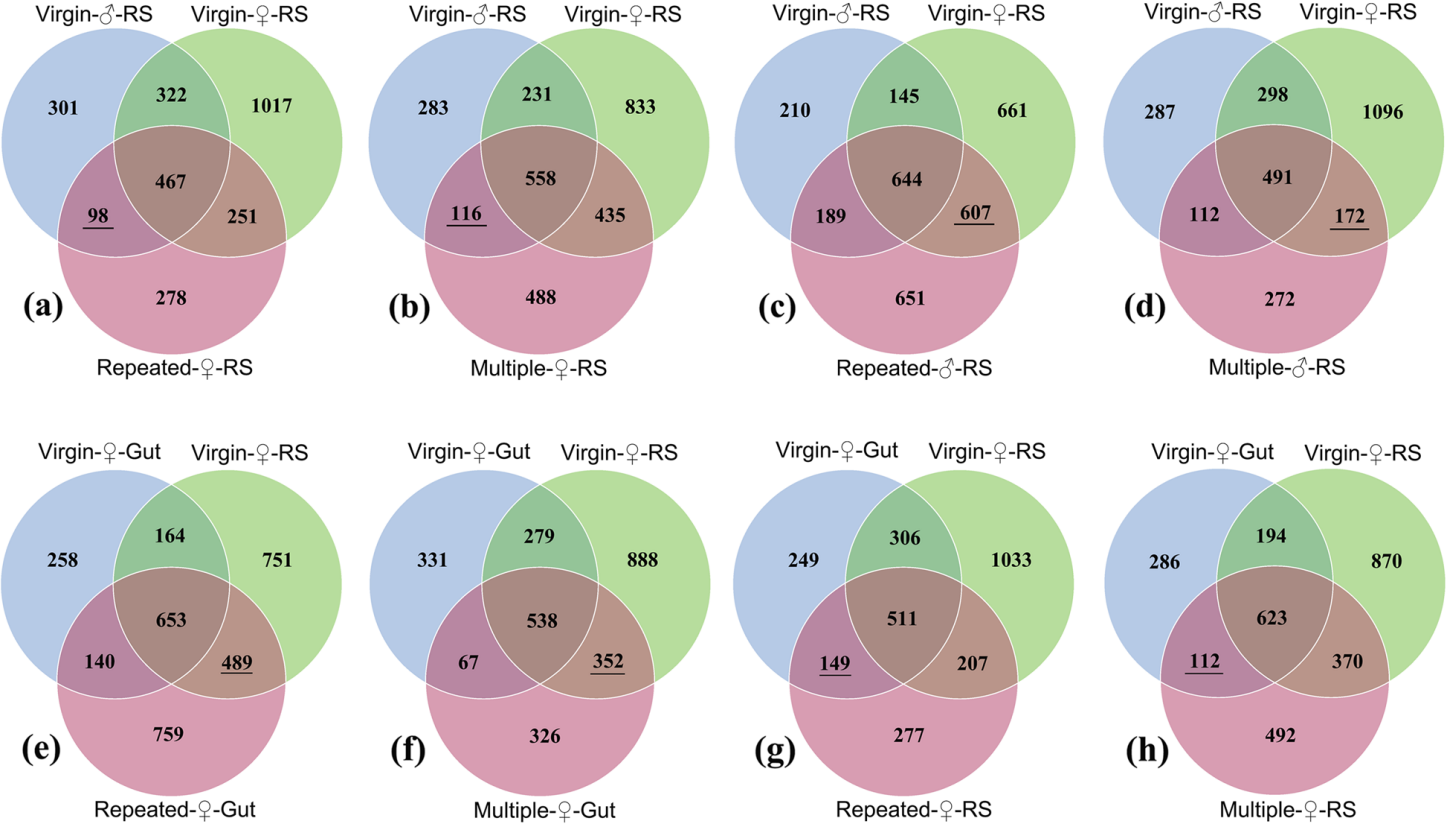
The OTU Venn diagrams of different samples from *S. frugiperda*. **a** Virgin-♂-RS vs. Virgin-♀-RS vs. Repeated-♀-RS; **b** Virgin-♂-RS vs. Virgin-♀-RS vs. Multiple-♀-RS; **c** Virgin-♂-RS vs. Virgin-♀-RS vs. Repeated-♂-RS; **d** Virgin-♂-RS vs. Virgin-♀-RS vs. Multiple-♂-RS; **e** Virgin-♀-Gut vs. Virgin-♀-RS vs. Repeated-♀-Gut; **f** Virgin-♀-Gut vs. Virgin-♀-RS vs. Multiple-♀-Gut; **g** Virgin-♀-Gut vs. Virgin-♀-RS vs. Repeated-♀-RS; and **h** Virgin-♀-Gut vs. Virgin-♀-RS vs. Multiple-♀-RS

There were 789, 657 and 619 common OTUs shared by Virgin-♂-RS and Virgin-♀-RS (Fig. S3a), Repeated-♂-RS and Repeated-♀-RS (Fig. S3b), and Multiple-♂-RS and Multiple-♀-RS (Fig. S3c); and 817, 642 and 705 common OTUs shared by Virgin-♀-Gut and Virgin-♀-RS (Fig. S3d), Repeated-♀-Gut and Repeated-♀-RS (Fig. S3e), and Multiple-♀-Gut and Multiple-♀-RS (Fig. S3f). Venn diagrams indicated that the RS of mated individuals harboured OTUs that were found in virgin individuals of the opposite sex but not in virgin individuals of the same sex: Repeated-♀-RS (Fig. 2a) and Multiple-♀-RS (Fig. 2b) had 98 and 116 such OTUs, respectively; Repeated-♂-RS (Fig. 2c) and Multiple-♂-RS (Fig. 2d) had more such OTUs at 607 and 172, respectively. Venn diagrams also indicated that the gut of mated individuals harboured OTUs that were found in the RS of virgin individuals (489 OTUs for Repeated-♀-Gut, Fig. 2e; and 352 OTUs for Multiple-♀-Gut, Fig. 2f) but not in the gut of virgin individuals, while the RS of mated individuals harboured OTUs that were found in the gut of virgin individuals (149 OTUs for Repeated-♀-RS, Fig. 2g; 112 OTUs for Multiple-♀-RS, Fig. 2h) but not in the RS of virgin individuals.

**Fig. 3:**
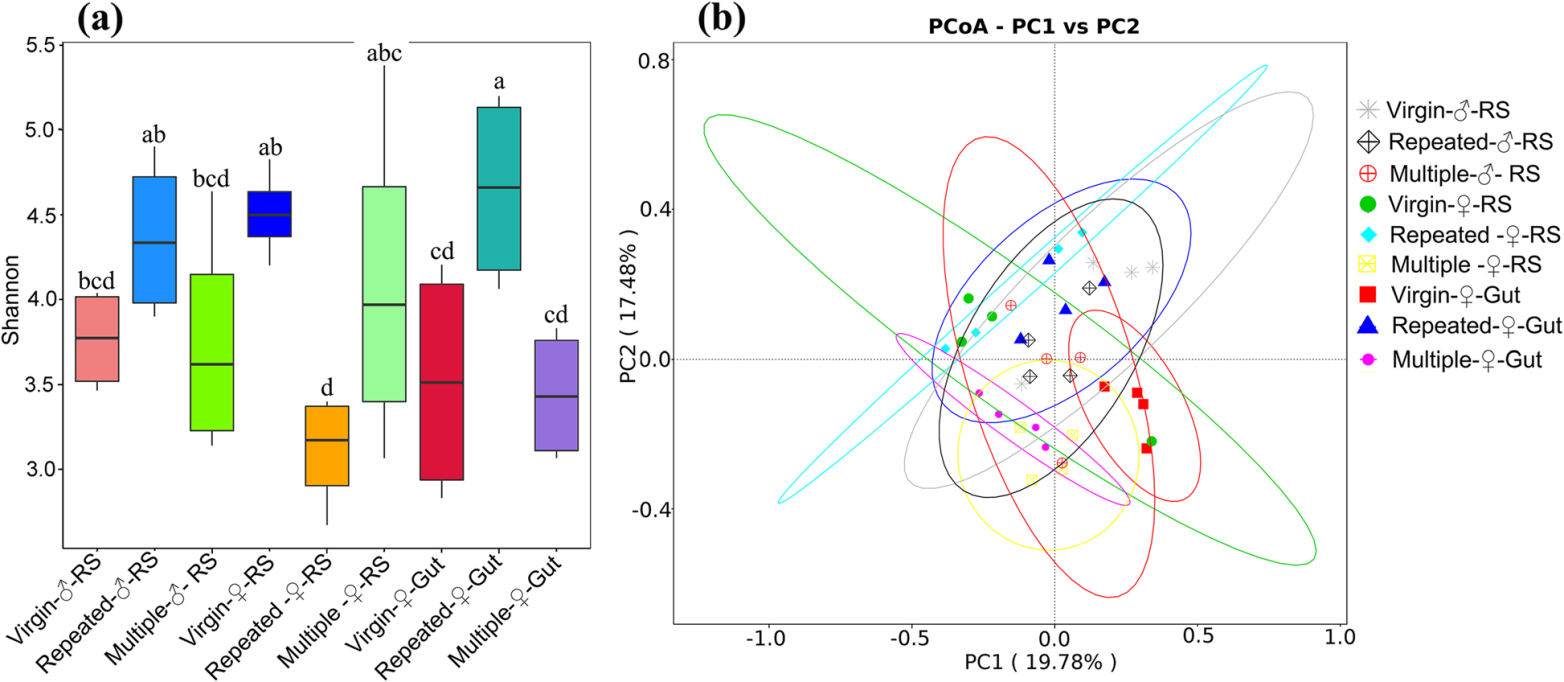
Shannon diversity indices (**a**) and PCoA ordination based on Bray–Curtis distances (**b**) of different samples from *S. frugiperda*. In subgraph (**a**), bars with different letters are significantly different (*P* < 0.05)

Alpha diversity indices from Shannon, Simpson and Chao1 suggested the variation of bacterial diversity between different tissues and different mating treatments (Table 1). Analysis of variance based on Shannon diversity metrics indicated that the OTU abundance was significantly different among all samples (ANOVA: *F*_1,34_ = 3.24, *P* = 0.010; Fig. 3a). A post hoc LSD test showed that Repeated-♀-Gut and Virgin-♀-RS had the highest abundance, followed by Repeated-♂-RS, Multiple-♀-RS, Virgin-♂-RS, Multiple-♂-RS, Virgin-♀-Gut and Multiple-♀-Gut, while Repeated-♀-RS had the lowest abundance (*P* < 0.05; Fig. 3a).

Beta diversity analysis based on the Bray–Curtis distance (illustrated by PcoA; Fig. 3b) further showed significant variances in the composition of OTUs among all samples (PERMANOVA: *F*_2,12_ = 2.339, *R*^*2*^ = 0.4093, *P* < 0.001). Pairwise comparisons within male RS of different mating treatments (Table S3a) revealed significant difference between Repeated-♂-RS and Virgin-♂-RS (*P* = 0.044), and marginally significant difference between Multiple-♂-RS and Virgin-♂-RS (*P* = 0.083). Pairwise comparisons within female RS of different mating treatments (Table S3b) revealed a significant difference between Repeated-♀-RS and Multiple-♀-RS (*P* = 0.035). Pairwise comparisons between male RS and female RS of virgin (Table S3c) or mated individuals (Table S3d) revealed significant difference between Virgin-♂-RS and Virgin-♀-RS (*P* = 0.022) and between Repeated-♂-RS and Repeated-♀-RS (*P* = 0.026) and a marginally significant difference between Repeated-♂-RS and Multiple-♀-RS (*P* = 0.087). Pairwise comparisons within the female gut of different mating treatments (Table S3e) revealed significant differences between Multiple-♀-Gut and Virgin-♀-Gut (*P* < 0.001), between Multiple-♀-Gut and Repeated-♀-Gut (*P* < 0.001), and between Virgin-♀-Gut and Repeated-♀-Gut (*P* = 0.028). Pairwise comparisons between the RS and gut of virgin females (Table S3f) or mated females (Table S3g) revealed a significant difference between Repeated-♀-RS and Multiple-♀-Gut (*P* = 0.030). Other pairwise comparison results are shown in Table S3.

### Taxonomy assignment

**Fig. 4:**
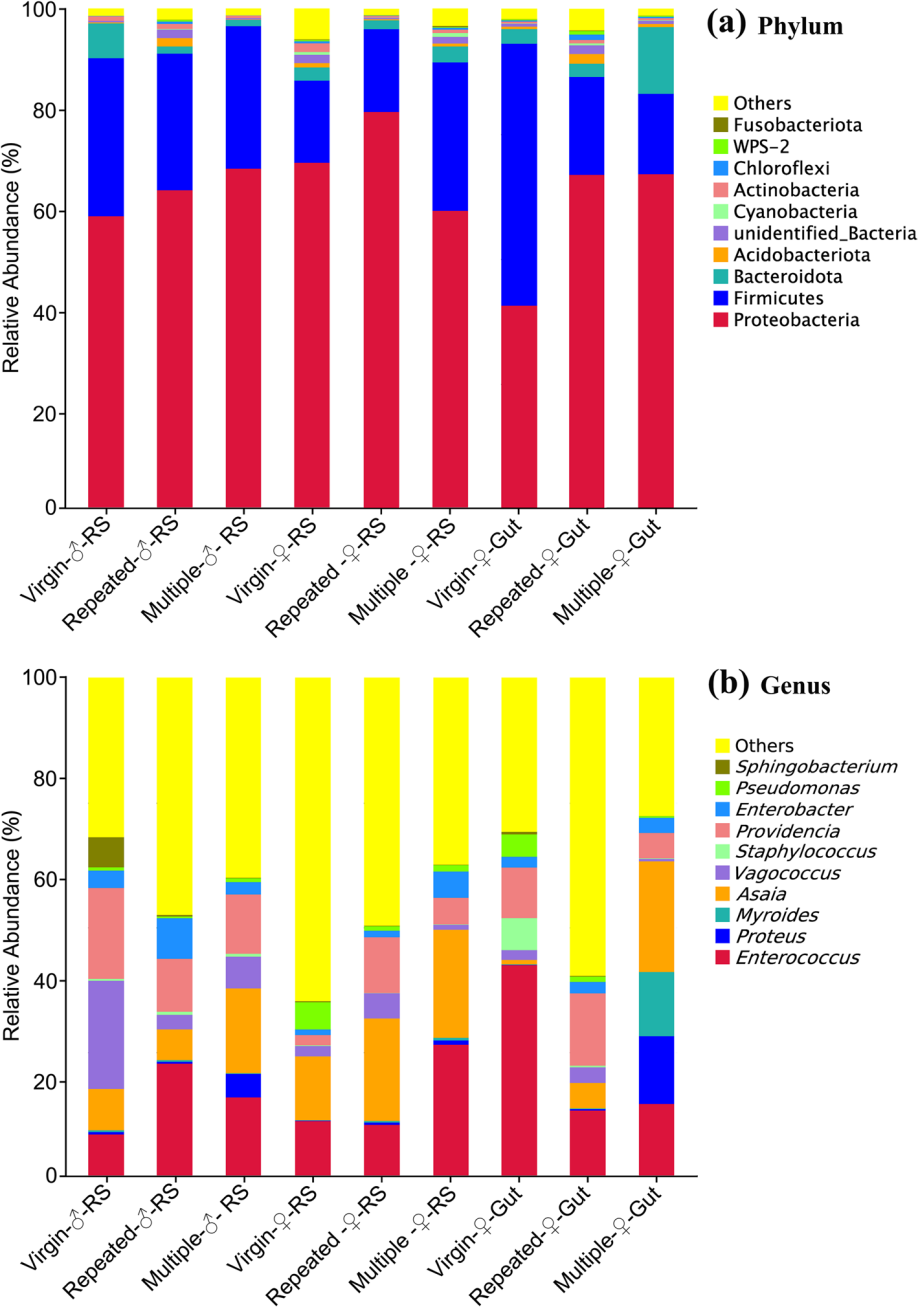
Taxonomic assignment of bacterial OTUs at the phylum (top 10; **a**) and genus (top 10; **b**) levels in different samples of *S. frugiperda*

The obtained 4315 OTUs (Table S2) were classified into 61 phyla (Table S4; Fig. 4a), 126 classes (Table S5), 271 orders (Table S6), 401 families (Table S7), 642 genera (Table S8; Fig. 4b) and 289 species (Table S9).

At the phylum level (Fig. 4a), Proteobacteria was the most predominant bacterial phylum in almost all samples (except Virgin-♀-Gut, Firmicutes), with virgin samples showing relatively lower abundance than mated samples within the male RS or female gut, while in female RS, Repeated-♀-RS showed the highest abundance, followed by Virgin-♀-RS and then Multiple-♀-RS. The second and third dominant phyla in most samples were Firmicutes and Bacteroidota (Fig. 4a). In contrast to Proteobacteria, the abundance of Firmicutes was relatively higher in virgin samples than in mated samples in the male RS or female gut, while in female RS, Multiple-♀-RS showed the highest abundance, followed by Repeated-♀-RS and then Virgin-♀-RS.

At the genus level (Fig. 4b), *Enterococcus* was the first dominant bacterial genus in Virgin-♀-Gut (42.72%), Multiple-♀-RS (26.75%) and Repeated-♂-RS (22.94%); *Asaia* was the first dominant genus in Multiple-♀-Gut (22.21%), Repeated-♀-RS (20.54%), Multiple-♂-RS (17.02%) and Virgin-♀-RS (12.82%); *Vagococcus* (21.68%) and *Providencia* (14.48%) were the first dominant genera of Virgin-♂-RS and Repeated-♀-Gut, respectively. Similar change patterns were observed in *Enterococcus* and *Asaia* within male or female RS, where they had a lower abundance in virgin samples but higher abundance in mated samples. Within the female gut, *Asaia* also showed a lower abundance in virgin samples but a higher abundance in mated samples; however, *Enterococcus* showed a much higher abundance in virgin samples but a lower abundance in mated samples.

LEfSe also showed remarkable differences in the number and taxa of microbial biomarkers between different samples (Fig. S4). The number of biomarkers ranged from 1 to 15 in different samples, with Repeated-♀-Gut, Multiple-♀-RS, Repeated-♀-RS and Virgin-♀-RS being ≥10 while others being ≤6; the taxa of microbial biomarkers were completely different between different samples.

### Functional prediction using FAPROTAX

**Fig. 5:**
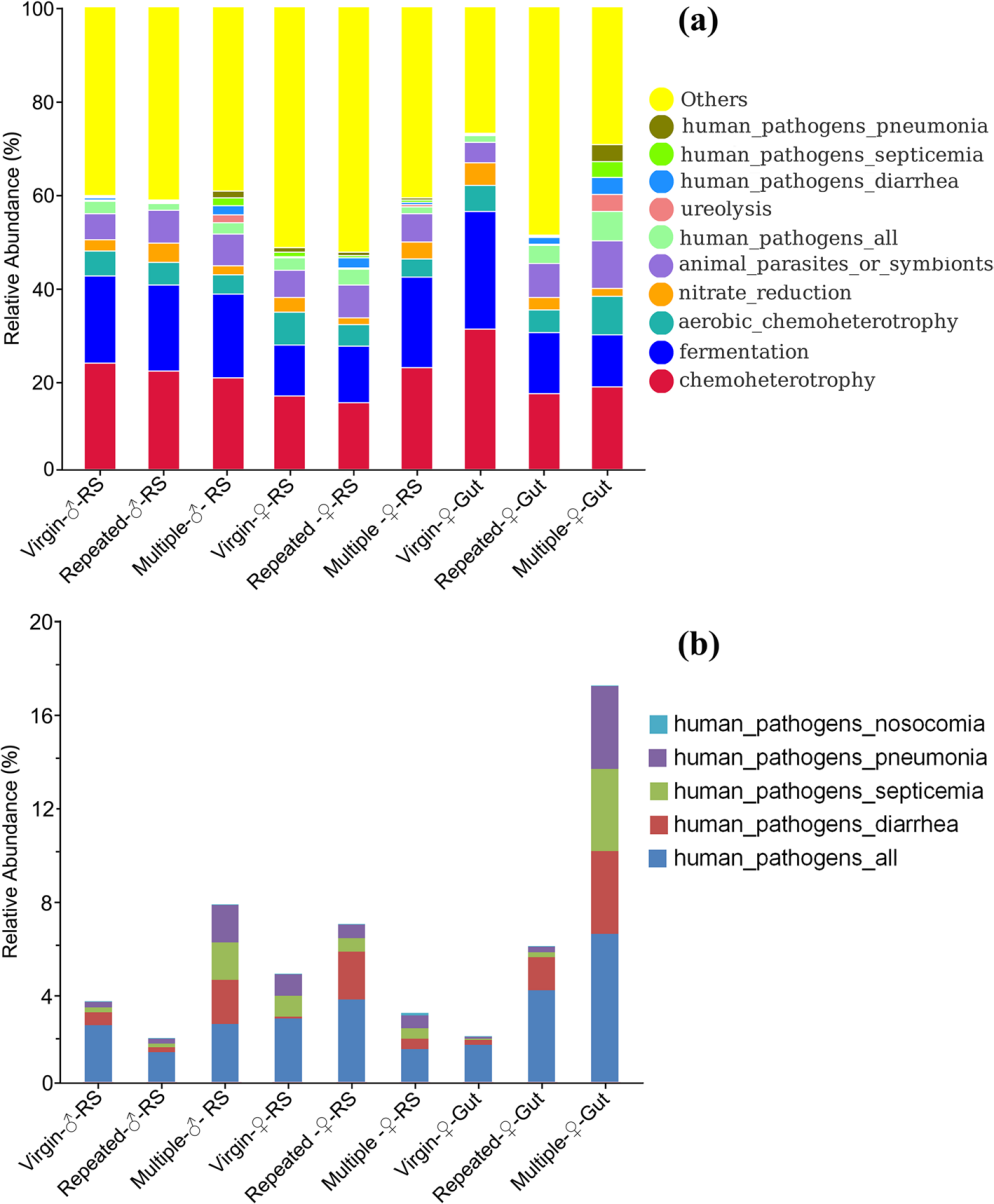
The relative abundance of dominant bacterial functional groups (top 10) (**a**) and the relative abundance of different pathogenic functions (**b**) of different samples from *S. frugiperda* (predicted by FAPROTAX)

FAPROTAX analysis (Fig. 5a; Fig. S5) showed that chemoheterotrophy (occupying 14.27 to 30.06% of the total annotated functions) and fermentation (10.92 to 25.58%) were the most dominant functions in all samples, with Virgin-♀-Gut showing the highest abundance in both functions. Mating also showed a remarkable effect on these two functions, where mating reduced their abundance in the male RS and female gut while increasing their abundance in the female RS.

As four of the top ten functions were related to human pathogens (Fig. 5a), we further grouped these functions and illustrated them in detail in Fig. 5b. the results (Fig. 5b) showed that mating largely increased the abundance of these functions in the female gut. In male RS, mating decreased them in Repeated-♂-RS but largely increased them in Multiple-♂-RS; while in female RS, mating increased them in Repeated-♀-RS but decreased them in Multiple-♀-RS.

### Pathogen and healthy bacterial profiles

**Fig. 6:**
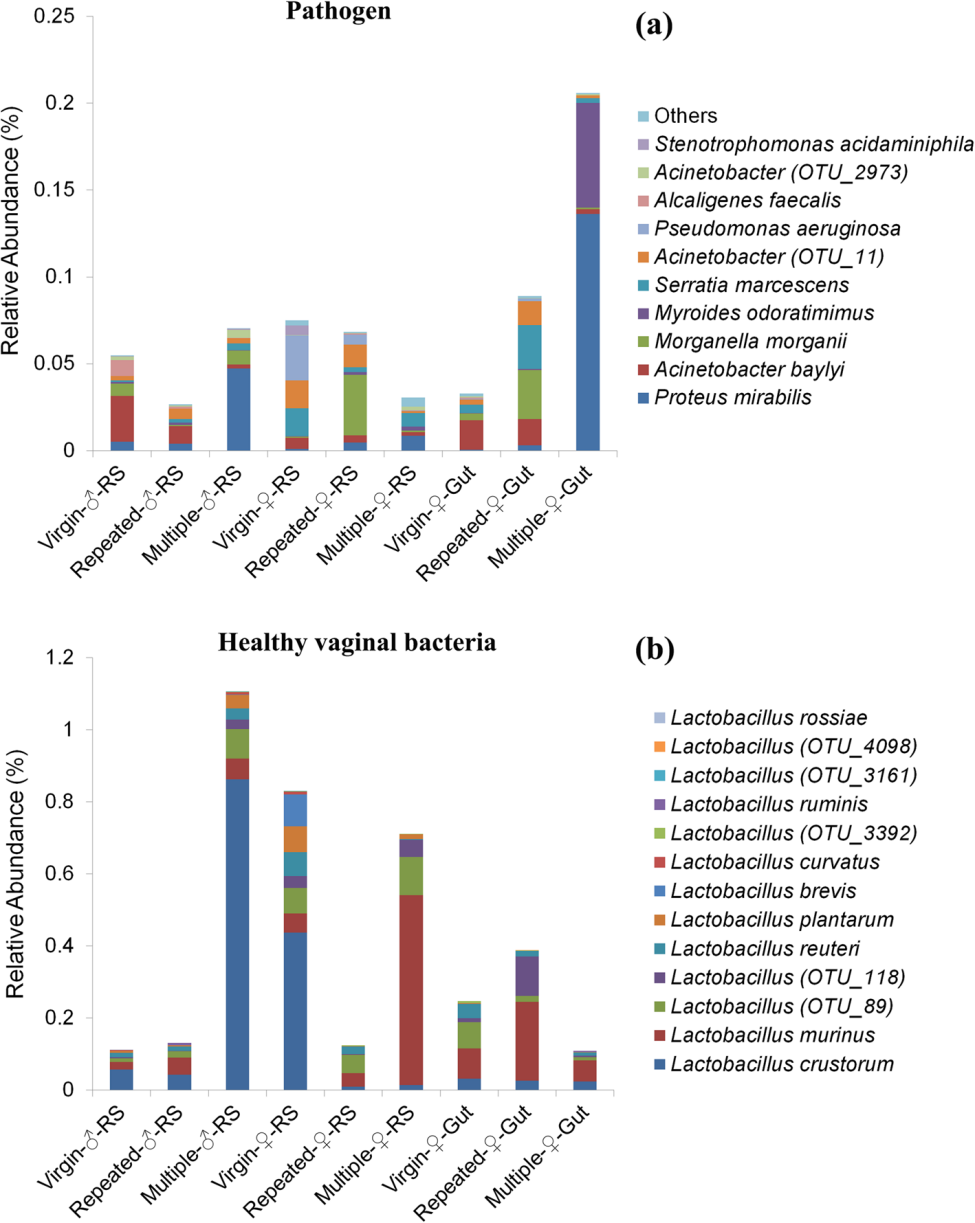
The relative abundance of possible pathogens (**a**) and healthy vaginal bacteria (**b**) in different samples of *S. frugiperda*

Based on the above taxonomy and functional prediction, the possible pathogens and healthy bacteria were further evaluated based on evidence from published studies. A total of 34 animal pathogens were found, including 3 pathogens of insects and others of other animals (Table 2). Statistical analysis showed that mating largely increased the abundance of these possible pathogens in the female gut (Fig. 6a), which was similar to the results of the functional test (Fig. 5b), while in RS, mated males or females might have decreased or increased the abundance of these pathogens (Fig. 6a).

**Table 2:** Possible pathogens and healthy vaginal bacteria profiles in *S. frugiperda*

Functional Group	Taxonomy			Functional Annotation	Reference
	Phylum	Order	Species		
Pathogen	Proteobacteria	Enterobacterales	*Morganella morganii*	Insect pathogen	[57]
	Proteobacteria	Enterobacterales	*Serratia marcescens*	Insect pathogen	[1, 58]
	Proteobacteria	Pseudomonadales	*Pseudomonas aeruginosa*	Insect pathogen	[58]
	Proteobacteria	Enterobacterales	*Serratia* spp. (OTU_1130; OTU_3075)	Animal pathogen	[1, 59]
	Proteobacteria	Acetobacterales	*Alcaligenes faecalis*	Human pathogen	[60]
	Proteobacteria	Acetobacterales	*Roseomonas* spp. (OTU_2563; OTU_3173; OTU_3212)	Human pathogen	[61]
	Proteobacteria	Burkholderiales	*Burkholderia cenocepacia*	Human pathogen	[62]
	Proteobacteria	Coxiellales	*Coxiella* spp. (OTU_2646; OTU_3289; OTU_3667; OTU_4069)	Human pathogen	[63]
	Proteobacteria	Enterobacterales	*Proteus mirabilis*	Human pathogen	[64, 65]
	Proteobacteria	Xanthomonadales	*Stenotrophomonas maltophilia*	Human pathogen	[66]
	Proteobacteria	Xanthomonadales	*Stenotrophomonas acidaminiphila*	Potential human pathogen	[67]
	Proteobacteria	Xanthomonadales	*Stenotrophomonas* spp. (OTU_3295)	Potential human pathogen	[68]
	Proteobacteria	Pseudomonadales	*Acinetobacter lwoffii*	Human and fish pathogen	[69, 70]
	Proteobacteria	Pseudomonadales	*Acinetobacter baylyi*	Human pathogen	[71]
	Proteobacteria	Pseudomonadales	*Acinetobacter ursingii*	Human pathogen	[72]
	Proteobacteria	Pseudomonadales	*Acinetobacter pittii*	Human pathogen	[73]
	Proteobacteria	Pseudomonadales	*Acinetobacter* spp. (OUT_11; OTU_1250; OTU_2772; OTU_2973; OTU_3124; OTU_3319; OTU_4025)	Human pathogen	[74]
	Firmicutes	Bacillales	*Bacillus anthracis*	Human pathogen	[75]
	Firmicutes	Lactobacillales	*Streptococcus anginosus*	Human pathogen	[76]
	Bacteroidota	Bacteroidales	*Bacteroides fragilis*	Human pathogen	[77]
	Bacteroidota	Flavobacteriales	*Empedobacter brevis*	Human pathogen	[78]
	Bacteroidota	Flavobacteriales	*Myroides odoratimimus*	Human pathogen	[79]
Healthy vaginal bacteria	Firmicutes	Lactobacillales	*Lactobacillus crustorum*	Healthy vaginal microbiome	[9, 10]
	Firmicutes	Lactobacillales	*Lactobacillus reuteri*	Healthy vaginal microbiome	[9, 10]
	Firmicutes	Lactobacillales	*Lactobacillus curvatus*	Healthy vaginal microbiome	[9, 10]
	Firmicutes	Lactobacillales	*Lactobacillus plantarum*	Healthy vaginal microbiome	[9, 10]
	Firmicutes	Lactobacillales	*Lactobacillus ruminis*	Healthy vaginal microbiome	[9, 10]
	Firmicutes	Lactobacillales	*Lactobacillus brevis*	Healthy vaginal microbiome	[9, 10]
	Firmicutes	Lactobacillales	*Lactobacillus murinus*	Healthy vaginal microbiome	[9, 10]
	Firmicutes	Lactobacillales	*Lactobacillus rossiae*	Healthy vaginal microbiome	[9, 10]
	Firmicutes	Lactobacillales	*Lactobacillus* spp. (OTU_3161; OTU_3392; OTU_4098; OTU_89; OTU_118)	Healthy vaginal microbiome	[9, 10]

Thirteen *Lactobacillus* spp. (healthy bacteria) were found in *S. frugiperda*, with *Lactobacillus crustorum* and *Lactobacillus murinus* often being the most dominant species in the samples (Table 2). Statistical analysis revealed a higher *Lactobacillus* abundance in the RS of virgin females but lower abundance in the RS of mated females (Fig. 6b). In contrast, *Lactobacillus* abundance was low in the RS of virgin males and increased markedly in the RS of multiple mated males (Fig. 6b).

## Discussion

Alpha diversity indices (Table 1; Fig. 3a) and beta diversity analysis (Fig. 3b), as well as LEfSe analysis revealed the variation in bacterial diversity between different samples, and showed that mating induced significant changes in the abundance and composition of the microbiota both in the RS and gut. The microbial composition and abundance in female reproductive organs is likely to be affected by the sexual transmission of microbes via male genitalia and the ejaculate and by microbes transferred from the cuticle to the reproductive organs during and after mating [80, 81]. A recent study in *C. lectularius* found that mating increased the similarity of the communities of male and female organs and mated individuals harboured bacteria that were found in nonmated individuals of the opposite sex but not in nonmated individuals of the same sex, suggesting that bacteria are sexually transmitted [25]. In the present study, we also found that the RS of mated individuals harboured OTUs that were found in virgin individuals of the opposite sex but not in virgin individuals of the same sex (Fig. 2a-d). However, we did not find that mating increased the similarity of the communities of male and female RS (Fig. S3a-c), which might be because mating differentially affected the microbiota diversity of male and female RS (Table 1). The vaginal microbiota may come from the gut or through a mother-to-child transfer [3], and the vaginal microbiota or invading bacteria may enter the haemolymph and other organs via copulatory wounds and the reproductive duct [18, 80, 81]. In the present study, Venn diagrams (Fig. 2e-h) also indicated that the gut of mated individuals harboured OTUs that were found in the RS of virgin individuals but not in the gut of virgin individuals, and the RS of mated individuals harboured OTUs that were found in the gut of virgin individuals but not in the RS of virgin individuals. These studies may partially suggest that mating induced microbiota transmission between male and female RS and between gut and RS. However, more direct evidence is needed.

The obtained 4315 OTUs were classified into 61 phyla (Table S4) and 642 genera (Table S8). Proteobacteria, Firmicutes and Bacteroidota are the top three dominant phyla either in male RS or female RS and gut in *S. frugiperda* (Fig. 4a), which is similar to the results obtained for the female abdomen of the same species [45]. Studies in other insect species also found that Proteobacteria was the predominant phylum in gut or reproductive organs, such as in the desert locust *Schistocerca gregaria* [82], the citrus fruit fly *Bactrocera minax* [26] and the sawfly *Cephalcia chuxiongica* [83]. Proteobacteria species may play important roles in insects, such as in helping insects fix nitrogen and preventing the proliferation and establishment of pathogens [3, 84–86]. At the genus level, however, the predominant genus is different in different samples; for example, *Enterococcus* is the most dominant genus in Virgin-♀-Gut, Multiple-♀-RS and Repeated-♂-RS, while *Asaia* is the most dominant genus in Multiple-♀-Gut, Repeated-♀-RS, Multiple-♂-RS and Virgin-♀-RS (Fig. 4b). These results further suggest that mating induces significant changes in the composition of the microbiota in both the RS and gut of *S. frugiperda*.

A previous study of the whole abdomen of female *S. frugiperda* showed that mating increased the abundance of pathogens or pathogenic functions [87]. In the present study, we further showed that matings dramatically increased the abundance of pathogens or pathogenic functions in the gut, while in the RS, the change pattern was not always consistent (Fig. 5; Fig. 6a). It is not clear why the abundance of pathogens increased in the gut of mated females. One possible reason for this phenomenon may be the trade-offs between reproduction and immunity, with enhanced reproductive activity limiting immune defence [28]. In this study*,* we paired males and females for mating after eclosion and collected samples 6 d after eclosion. Mated females usually lay most (approximately 70%) of their egg load at 6 d of age [48]. From the whole-body perspective, mating seems to have reduced the immune activity in *S. litura* [88]. The inconsistent change patterns in female RS may be because mating increased regional immune activity due to male-transferred foreign materials and infections [30, 31, 89, 90].

Taxonomy assignment revealed thirteen *Lactobacillus* spp. in *S. frugiperda*, with *Lactobacillus crustorum* and *Lactobacillus murinus* presenting a high abundance in most samples (Fig. 6b). Moreover, we found that the *Lactobacillus* spp. showed a higher abundance in the RS of virgin females and a lower abundance in the RS of virgin males and the gut of virgin females (Fig. 6b). However, mating reduced the abundance of *Lactobacillus* spp. in the RS of females, while their abundance increased in the RS of males, especially in males mated with multiple females (Fig. 6b). More importantly, the RS of virgin females and the RS of multiple mated males were very similar in terms of the composition and abundance of different *Lactobacillus* species, with *Lactobacillus crustorum* showing much higher abundance in both the RS of virgin females and the RS of males mated with multiple females (Fig. 6b). The reverse change patterns of these species in male and female RS suggest that these bacterial strains are sexually transmitted between sexes in *S. frugiperda*.

*Lactobacillus* spp. are Gram-positive bacilli, which are believed to originate from the gut [91]. Consistently, *Lactobacilli* can also be found in the gut of female *S. frugiperda* (Fig. 6b). In humans, *Lactobacilli* are dominant vaginal microbiota but also are abundant in the gut [92]. *Lactobacilli* in the gut may play multiple functions, such as energy metabolism and physiological and immunologic homeostasis [87, 92]. In the vagina, *Lactobacilli* produced lactate plays an important role in maintaining a low vaginal pH (3.5–4.5), which is crucial to prevent the colonization and development of opportunistic pathogens including those that may translocate from the gut or from males [91, 93, 94]. A number of *Lactobacillus* strains, such as *L. rhamnosus* GR-1, *L. reuteri* RC-14, *L. brevis* CD2 and *L. salivarius* FV2, have been used orally or intravaginally in the treatment of bacterial vaginosis [95, 96]. Three species found in *S. frugiperda*, namely *L. reuteri*, *L. plantarum* and *L. brevis* (Table 2), have also been identified as human ‘healthy’ vaginal bacterial species [3]. However, whether these *Lactobacillus* spp. also benefit female moths is still unknown and thus warrants further study. Future studies on the microbiome and metabolite profile interactions and comparative analysis across different taxa from the perspective of evolution and ecosystems are expected to provide deeper insights in this field.

## Conclusion

Mating resulted in significant changes in the abundance and composition of the microbiome in the RS and gut of *S. frugiperda*. Bacterial OTUs were classified into 61 phyla and 642 genera, with Proteobacteria, Firmicutes and Bacteroidota being the most predominant phyla either in male RS or female RS and gut. *Enterococcus* and *Asaia* were the dominant genera in most samples. Mating increased the abundance of pathogens or pathogenic functions in the gut, whereas the change range was trivial in the RS. A total of thirteen *Lactobacillus* spp. were found in *S. frugiperda*, with *Lactobacillus crustorum* and *Lactobacillus murinus* showing high abundance. Three *Lactobacillus* species found in *S. frugiperda*, *L. reuteri*, *L. plantarum* and *L. brevis*, have also been identified as the human “healthy” vaginal microbiome. *Lactobacillus* spp. showed higher abundance in the RS of virgin females and lower abundance in the RS of virgin males and the gut of virgin females. Mating reduced their abundance in the RS of females, but increased their abundance in the RS of males, especially in males mated with multiple females. The abundance and composition of *Lactobacillus* species in the RS of multiple mated males are very similar to those of the RS of virgin females, suggesting that these bacteria are transferred from female RS to male RS through mating. The higher abundance of *Lactobacillus* spp. in the RS of female moths and the similarity of *Lactobacillus* species in *S. frugiperda* with human ‘healthy’ vaginal *Lactobacillus* spp. suggest that these bacterial strains may also play a similar role in the reproductive tract of female moths.

## Supplementary Information

**Additional file 1: Table S1.** Summary of the quality of all samples’ sequencing data. **Table S2.** OTU relative abundance of all samples. **Table S3.** Pairwise comparisons based on Bray-Curtis distance. **Table S4.** OTUs classified into phylum. **Table S5.** OTUs classified into class. **Table S6.** OTUs classified into order. **Table S7.** OTUs classified into family. **Table S8.** OTUs classified into genus. **Table S9.** OTUs classified into species.

**Additional file 2: Fig. S1.** Rarefaction curves represent observed OTUs in different samples of *S. frugiperda*. **Fig. S2.** The petals diagram of OTUs from different samples of *S. frugiperda*. **Fig. S3.** The Venn diagrams of OTUs from different samples of *S. frugiperda*. (a) Virgin-♂-RS vs Virgin-♀-RS; (b) Repeated-♂-RS vs Repeated-♀-RS; (c) Multiple-♂-RS vs Multiple-♀-RS; (d) Virgin-♀-Gut vs Virgin-♀-RS; (e) Repeated-♀-RS vs Repeated-♀-Gut; and (f) Multiple-♀-RS vs Multiple-♀-Gut. **Fig. S4.** Important biomarks of bacterial communities in different samples of *S. frugiperda* revealed by linear discriminant analysis (LDA). **Fig. S5.** The heat map of bacterial functions (top 25) from the FAPROTAX database for *S. frugiperda* samples. The samples are grouped according to the similarity of each other. Different colors indicate the relative abundance of groups in the individual samples, wherein red represents the function with higher abundance, and blue represents the function with lower abundance in the corresponding sample.

## Data Availability

The raw reads from 16S rDNA gene sequencing were deposited into the NCBI (https://www.ncbi.nlm.nih.gov/) Sequence Read Archive (SRA) database, the login number is PRJNA844607. Other data generated or analysed during this study are included in this article and its Supplementary Information files.
