# Supplementary material for: Lactobacillus spp. in the reproductive system of female moths and mating induced changes and possible transmission
Source: BMC Microbiol. 2022 Dec 19;22:308. doi: 10.1186/s12866-022-02724-6 (PMC9762107; doi:10.1186/s12866-022-02724-6)
Supplement: Supplementary file 2 — Additional file 2: Fig. S1. Rarefaction curves represent observed OTUs in different samples of S. frugiperda. Fig. S2. The petals diagram of OTUs from different samples of S. frugiperda. Fig. S3. The Venn diagrams of OTUs from different samples of S. frugiperda. (a) Virgin-♂-RS vs Virgin-♀-RS; (b) Repeated-♂-RS vs Repeated-♀-RS; (c) Multiple-♂-RS vs Multiple-♀-RS; (d) Virgin-♀-Gut vs Virgin-♀-RS; (e) Repeated-♀-RS vs Repeated-♀-Gut; and (f) Multiple-♀-RS vs Multiple-♀-Gut. Fig. S4. Important biomarks of bacterial communities in different samples of S. frugiperda revealed by linear discriminant analysis (LDA). Fig. S5. The heat map of bacterial functions (top 25) from the FAPROTAX database for S. frugiperda samples. The samples are grouped according to the similarity of each other. Different colors indicate the relative abundance of groups in the individual samples, wherein red represents the function with higher abundance, and blue represents the function with lower abundance in the corresponding sample. [file 12866_2022_2724_MOESM2_ESM.pdf]

Lactobacillus spp. in the reproductive system of female moths and mating induced changes and possible transmission

Short running title: **_Lactobacillus_ spp. in moths**

Qing-Yi Zhao[1,2], Luo-Yan Zhang[1,*], Da-Ying Fu[1], Jin Xu[1,2,*], Peng Chen[3], Hui Ye[4]

[1]Yunnan Academy of Biodiversity, Southwest Forestry University, Kunming 650224, China

[2]Key Laboratory for Forest Resources Conservation and Utilization in the Southwest Mountains of China, Ministry of Education, Southwest Forestry University, Kunming 650224, China

[3]Yunnan Academy of Forestry and Grassland, Kunming 650201, China

[4]School of Ecology and Environment, Yunnan University, Kunming 650091, China

[*]Correspondence: Luo-Yan Zhang, Email: 1634746839@qq.com; Jin Xu, Email: xujin2798@126.com

Abstract

Background: The microbiome in the insect reproductive tract is poorly understood. Our previous study demonstrated the presence of *Lactobacillus* spp. in female moths, but their distribution and function remain unclear. *Lactobacillus* spp. are known as the 'healthy' vaginal microbiome in humans.

Results: Here, we studied the microbiome in the reproductive system (RS) and gut of *Spodoptera frugiperda* using 16S rDNA sequences. The obtained 4315 bacterial OTUs were classified into 61 phyla and 642 genera, with Proteobacteria, Firmicutes and Bacteroidota being the top three dominant phyla and *Enterococcus* and *Asaia* being dominant genera in most samples. Mating dramatically increased the abundance of pathogens or pathogenic functions in the gut, while in the RS, the change range was trivial. Taxonomy assignment identified thirteen *Lactobacillus* spp. in *S. frugiperda*, with *Lactobacillus crustorum* and *Lactobacillus murinus* showing high abundance. Three species found in *S. frugiperda,* namely *L. reuteri*, *L. plantarum* and *L. brevis*, have also been identified as human 'healthy' vaginal bacterial species. *Lactobacillus* spp. showed higher abundance in the RS of virgin females and lower abundance in the RS of virgin males and the gut of virgin females. Mating reduced their abundance in the RS of females but increased their abundance in the RS of males, especially in males mated with multiple females. The RS of virgin females and of multiple mated males were very similar in terms of composition and abundance of *Lactobacillus* species, with *Lactobacillus crustorum* showing much higher abundance in both tissues, potentially due to sexual transmission.

Conclusions: *Lactobacillus* spp. showed high abundance and diversity in the RS of female moths. The higher abundance of *Lactobacillus* spp. in the RS of female moths and the similarity of *Lactobacillus* species in female moths with human 'healthy'

vaginal *Lactobacillus* spp. suggest that these bacterial strains are also an important microbiome in the RS of female moths.

Key words: *Spodoptera frugiperda*; *Lactobacillus*; Reproductive system; Gut; Mating; Sexually transmitted microbes; Healthy vaginal microbiome

Background

To date, a large number of studies have been conducted to reveal the role of the gut microbiome in different animals and have provided deep insight from the perspectives of ecology, adaptation and evolution [1-5]. These studies have shown that gut microbes may contribute to the host by providing nutritional components, promoting digestive efficiency, and helping the host to live on a suboptimal environmental diet. For example, nitrogen fixation by gut microbiota in some termites can account for up to 60% of the nitrogen obtained by the host [6]. In the human gut, bacterial fermentation products, such as short-chain fatty acids, have been shown to play anti-inflammatory and protective roles [3]. Resident gut bacteria may also benefit insects by providing protection against colonization of the gut by pathogens [7] or by counteracting plant toxic defences [8].

Studies in mammals have revealed a diversity of microbial residents in reproductive organs and sexually transmitted microbes [9, 10]. Some of these microbes may have significant effects on the survival and reproductive success of males and females [9, 10]. Studies in male mammals have recognized that some of the sexually transmitted microbes have negative effects on sperm quality and fertility [9-11]. Evidence from female mammals increasingly recognizes that some of these microbes are associated with female reproduction, such as infertility, preterm birth and sexually transmitted diseases [10, 12-15]. Moreover, studies in humans have also identified some ‘healthy’ vaginal microbes, such as *Lactobacillus* spp., which are typically the dominant vaginal microbes. These ‘healthy’ microbes are oxygen-tolerant anaerobes and exhibit antimicrobial activity against a range of vaginal pathogens, probably by producing of lactic acid, stimulating host defence responses and physically barring against pathogen adhesion [13, 14, 16, 17]. However, the microbiota in insect

reproductive organs is relatively less well known [9, 18]. Evidence for such reproduction-related pathogens or healthy microbes is especially limited in insects. A study by Otti et al. [19] showed that exposing bedbugs to polymicrobial mixtures (including *Bacillus*, *Staphylococcus*, *Acinetobacter* and *Alcaligenes*) resulted in high sperm mortality (up to 40%). Studies in fruit flies have also demonstrated that some *Enterococcus* species, such as *E. faecalis,* may have a negative impact on the fecundity of the host [20, 21]. However, no such 'healthy' vaginal microbes have been identified in insects thus far.

Furthermore, studies in humans have demonstrated that the microbiome of the female gut and reproductive organs represent very complex biological ecosystems; they communicate with each other and cause widespread impacts on the host, such as functions in host immunological and metabolic homeostasis [3]. These vaginal microbes may derive from the gut by continuous translocation and have evolved to adapt to the vaginal microenvironment [22, 23]. However, the functions elicited by these microbes and their metabolic byproducts within reproductive organs seem to be different from those in the gut [3]. For example, bacterial short-chain fatty acids play anti-inflammatory and protective roles in the gut, while they may exhibit dysbiotic and proinflammatory effects in the genital tract [3, 24]. Therefore, study of the gut and genital tract microbiome-induced crosstalk is helpful to achieve new findings in this field.

One possible reason for the limited evidence on such a reproduction-related microbiome in insects is that most of these microbes are uncultivable [18]. Recent developments in high-throughput sequencing and bioinformatics have provided an effective approach for understanding the symbiotic microbiome. Studies in a few insects have revealed the bacterial communities in their reproductive organs by using

16S rDNA sequencing and bioinformatic analysis [18, 25-27]. Sequencing and analysis in the Chinese citrus fly *Bactrocera minax* revealed that the female ovary has a higher diversity of the microbiome than the male testis, and the bacterial diversity of reproductive organs is higher than that of the gut [26]. Bellinvia et al. [18, 25] recently reported that the genital microbiome is sexually transmittable between sexes and may play roles in shaping the evolution of reproductive traits [18, 25]. However, no studies have tested the effect of mating on the microbiota both in the gut and reproductive organs.

Mating may have a significant (negative or positive) effect on female immunity [28, 29], which may also affect the abundance and diversity of the reproduction-related microbiome. Mating may negatively affect female immune activity due to trade-offs between reproduction and survival [28] and males may directly suppress female immunity to promote sperm storage and egg fertilization [29]. Studies have also indicated that mating may upregulate the female immune response due to the transfer of foreign materials and mating induced infections [30-33]. Females of polygamous species thus may have higher post-mating immunity if sexually transmitted infection is the major factor driving female postmating costs [31]. Sexually transmitted infection may negatively affect female lifespan and reproductive output in insects [34]. Therefore, it will be interesting and informative to study the reproductive microbiome in different mating systems under different mating conditions.

The fall armyworm (*Spodoptera frugiperda*) is currently a major worldwide agricultural pest, that mainly attacks corn, rice, wheat, sorghum and cotton [35]. This moth pest is native to tropical and subtropical regions in the Americas [36]. It was first discovered in the southwestern China at the end of 2018, and then spread to vast areas of China soon after [37]. This pest is notorious due to its long-distance migration

ability [38], high fecundity [39] and strong pesticide resistance [40, 41]. Environmentally friendly and sustainable management strategies, such as modifying microbial communities [42-44], are required for better control of this pest in the future.

Our previous study in *S. frugiperda* using the whole abdomen of female adults provided interesting results, wherein mating caused a decline in the diversity of symbiotic microbiomes and promiscuity incurred a higher pathogen abundance [45]. In the present study, we further studied the diversity and abundance of the microbiome in the reproductive system (both male and female) and gut (female) of *S. frugiperda*, and mating caused changes and transmissions. The evolutionary significance and crosstalk between the gut and reproductive organ microbiomes and between the host and microbiome are explored and discussed.

Materials and methods

Insect rearing and sampling

The larvae of *S. frugiperda* were collected in corn fields near Dongchuan town in Yunnan Province, China. The larvae were reared on an artificial diet [46] under 28 ± 1 °C and 60-80% relative humidity with a 14:10-h light:dark photoperiod. Adults were fed a 10% honey solution. The offspring were used in the present study.

To ensure virginity and age, male and female pupae were sexed based on morphological characteristics [47] and then caged separately. Newly eclosed adults were collected and used for the following treatment and sampling. To sample the male reproductive system (RS) and female RS and gut under different mating conditions, five treatments were established (Fig. 1a): (1) virgin males were individually caged from the day of eclosion until sampling, and their RS were sampled to obtain the sample of Virgin-♂-RS; (2) virgin females were individually caged from the day of

eclosion until sampling, and their RS and gut were sampled to obtain Virgin-♀-RS and Virgin-♀-Gut, respectively; (3) virgin males and females were paired permanently with one pair per box from the second day since eclosion until sampling, and males and females mated at least two times with the same mates were used for RS and gut sampling to obtain the Repeated-♂-RS, Repeated-♀-RS and Repeated-♀-Gut; (4) a virgin male was provided with one novel age-matched virgin female each night from the second day since eclosion until sampling, and males mated at least two times with different females were used for RS sampling to obtain Multiple-♂-RS; and (5) a virgin female was provided with one novel age-matched virgin male each night from the second day since eclosion until sampling, and females mated at least two times with different males were used for RS and gut sampling to obtain Multiple-♀-RS and Multiple-♀-Gut. Males and females were paired during the night but separated during the daytime. The mating events (Fig. 1b) of all pairs were recorded daily by quickly observing treated insects every 30 min (the mating duration was approximately 1 h [48]). The experimental conditions were the same as described above. Each box was supplied with a 10% honey solution as food. A 15 W red light was used for illumination during observation. After the mating treatments, the male RS and female RS and gut were sampled at the end of the 6th day after eclosion through dissection. The males and females that were mated repeatedlly mated two to five times before sampling had mating times of 2.72 ± 0.07 (mean ± SE). The multiply mated males or females, which were also mated two to five times, had mating times of 3.02 ± 0.06 and 2.83 ± 0.06, respectively. Whole bodies of males or females were rinsed twice with sterile water, surface-sterilized in 75% ethanol for 90 s, and then rinsed twice again using sterile water. The moths were dissected to obtain the male RS (Fig. 1c) or female RS (Fig. 1d) and gut (Fig. 1e) in a plate containing 10 ml sterile PBS buffer (pH 7.4)

under a stereomicroscope. To obtain sufficient tissue for sequencing, 30 RS or gut (from 30 moths) were combined to form a sample replicate. Four biological replicates were used for each sample (n = 4). The samples were stored at −80 °C until use.

DNA extraction and 16S rDNA sequencing

The CTAB method [49] was used to extract total genomic DNA from samples, and 1.0% agarose gel electrophoresis was used to examine the DNA purity and concentration. The 16S rDNA regions were amplified via PCR using Phusion High-Fidelity PCR Master Mix (New England Biolabs, USA) and the V3-V4 primers (515F: 5′-GTGYCAGCMGCCGCGGTAA-3′; 806R: 5′-GGACTACNNGGGTATCTAAT-3′). The PCR products were analysed using 2% agarose gel electrophoresis and purified with a GeneJET Gel Extraction Kit (Thermo Scientific, USA). Sequencing libraries were prepared using a TruSeq DNA PCR-Free Library Preparation Kit (Illumina, USA) following the manufacturer's protocol. The library quality was assessed using a Qubit Fluorometer (Thermo Scientific, USA) and an Agilent Bioanalyzer 2100 system (Agilent Technologies, USA). Finally, the library was submitted for 16S rDNA sequencing using the Illumina NovaSeq PE250 platform at Beijin Novogene Bioinformatics Technology Co., Ltd.. The obtained raw data were deposited into the NCBI SRA database (Project No.: PRJNA844607).

Data analysis

Raw sequencing reads were assembled to obtain raw tags using FLASH software (v1.2.7, http://ccb.jhu.edu/software/FLASH/) [50]. Subsequently, clean tags were obtained after filtering low-quality and short-length raw tags with fastp v0.23.0 [51]. The effective tags were obtained by filtering the chimeric sequences in the raw tags

using VSEARCH software (v2.19.0, https://github.com/torognes/vsearch/) [52]. The values of Q20 and Q30 of the effective tags were calculated to evaluate the sequence quality. Effective tags were analysed using UPARSE software (UPARSE v7.0.1001, http://drive5.com/uparse/) [53]. Sequences with ≥ 97% similarity were assigned to the same OTUs. A representative sequence for each OTU was screened for subsequent annotation by the Silva Database (https://www.arb-silva.de/) [54] based on the Mothur algorithm.

Alpha diversity was performed to reveal the complexity of communities within samples using QIIME (Version 1.7.0). Alpha diversity indices, including Observed species, Shannon, Simpson, Chao1 and Good's coverage indices, were calculated. The differences in Shannon indices between samples were further analysed using ANOVA followed by LSD tests with Benjamini–Hochberg correction for multiple comparisons. Beta diversity was applied to assess the differences in the microbial community between samples. The significance of differences among or between samples was tested by permutational multivariate analysis of variance (PERMANOVA) based on Bray–Curtis metrics and then visualized accordingly by principal coordinates analysis (PCoA). PERMANOVA was performed using R (Version 4.0.3) with the vegan and phyloseq packages.

Linear discriminant analysis of effect size (LEfSe) (http://huttenhower.sph.harvard.edu/galaxy/) was used to determine OTUs that discriminate among the populations with an LDA score greater than 3.0.

Functional annotation of prokaryotic taxa (FAPROTAX) [55] was used to predict the potential functional annotation of bacterial taxa in different samples, which predicts functions of uncultured prokaryotes from the known functions of cultured bacterial

genera. FAPROTAX is a promising tool for predicting ecologically relevant functions of bacterial and archaeal taxa derived from 16S rDNA amplicon sequencing [56].

Results

Sequencing and quality control

By 16S rDNA sequencing, ~82 300 effective tags were obtained from each of the 36 libraries, with an average length of 417-428 bp (Table S1). The percentages of Q20 and Q30 of all samples' effective tags were 98.42-99.24% and 94.74-96.95%, respectively, indicating that the assembly quality was good. These effective tags were clustered into 4315 OTUs (Table S2). Rarefaction analysis showed a saturating number of OTUs (Fig. S1), indicating an adequate sequencing output for all samples.

Diversity indices of bacterial OTUs

The Good's coverages were all greater than 99% for all samples (Table 1), suggesting that the number of clones sampled was sufficient to provide an adequate estimation of bacterial diversity in *S. frugiperda*. The number of OTUs in different samples ranged from 1047 to 2091 (Table 1). Approximately 5% (216/4315%) of OTUs were shared by all samples (Fig. S2).

There were 789, 657 and 619 common OTUs shared by Virgin-♂-RS and Virgin-♀-RS (Fig. S3a), Repeated-♂-RS and Repeated-♀-RS (Fig. S3b), and Multiple-♂-RS and Multiple-♀-RS (Fig. S3c); and 817, 642 and 705 common OTUs shared by Virgin-♀-Gut and Virgin-♀-RS (Fig. S3d), Repeated-♀-Gut and Repeated-♀-RS (Fig. S3e), and Multiple-♀-Gut and Multiple-♀-RS (Fig. S3f). Venn diagrams indicated that the RS of mated individuals harboured OTUs that were found in virgin individuals of the opposite sex but not in virgin individuals of the same sex:

Repeated-♀-RS (Fig. 2a) and Multiple-♀-RS (Fig. 2b) had 98 and 116 such OTUs, respectively; Repeated-♂-RS (Fig. 2c) and Multiple-♂-RS (Fig. 2d) had more such OTUs at 607 and 172, respectively. Venn diagrams also indicated that the gut of mated individuals harboured OTUs that were found in the RS of virgin individuals (489 OTUs for Repeated-♀-Gut, Fig. 2e; and 352 OTUs for Multiple-♀-Gut, Fig. 2f) but not in the gut of virgin individuals, while the RS of mated individuals harboured OTUs that were found in the gut of virgin individuals (149 OTUs for Repeated-♀-RS, Fig. 2g; 112 OTUs for Multiple-♀-RS, Fig. 2h) but not in the RS of virgin individuals.

Alpha diversity indices from Shannon, Simpson and Chao1 suggested the variation of bacterial diversity between different tissues and different mating treatments (Table 1). Analysis of variance based on Shannon diversity metrics indicated that the OTU abundance was significantly different among all samples (ANOVA: $F_{1,34} = 3.24$, $P = 0.010$; Fig. 3a). A post hoc LSD test showed that Repeated-♀-Gut and Virgin-♀-RS had the highest abundance, followed by Repeated-♂-RS, Multiple-♀-RS, Virgin-♂-RS, Multiple-♂-RS, Virgin-♀-Gut and Multiple-♀-Gut, while Repeated-♀-RS had the lowest abundance ($P < 0.05$; Fig. 3a).

Beta diversity analysis based on the Bray–Curtis distance (illustrated by PcoA; Fig. 3b) further showed significant variances in the composition of OTUs among all samples (PERMANOVA: $F_{2,12} = 2.339$, $R^2 = 0.4093$, $P < 0.001$). Pairwise comparisons within male RS of different mating treatments (Table S3a) revealed significant difference between Repeated-♂-RS and Virgin-♂-RS ($P = 0.044$), and marginally significant difference between Multiple-♂-RS and Virgin-♂-RS ($P = 0.083$). Pairwise comparisons within female RS of different mating treatments (Table S3b) revealed a significant difference between Repeated-♀-RS and Multiple-♀-RS ($P = 0.035$). Pairwise comparisons between male RS and female RS of virgin (Table S3c) or mated

individuals (Table S3d) revealed significant difference between Virgin-♂-RS and Virgin-♀-RS (P = 0.022) and between Repeated-♂-RS and Repeated-♀-RS (P = 0.026) and a marginally significant difference between Repeated-♂-RS and Multiple-♀-RS (P = 0.087). Pairwise comparisons within the female gut of different mating treatments (Table S3e) revealed significant differences between Multiple-♀-Gut and Virgin-♀-Gut ($P < 0.001$), between Multiple-♀-Gut and Repeated-♀-Gut ($P < 0.001$), and between Virgin-♀-Gut and Repeated-♀-Gut (P = 0.028). Pairwise comparisons between the RS and gut of virgin females (Table S3f) or mated females (Table S3g) revealed a significant difference between Repeated-♀-RS and Multiple-♀-Gut (P = 0.030). Other pairwise comparison results are shown in Table S3.

Taxonomy assignment

The obtained 4315 OTUs (Table S2) were classified into 61 phyla (Table S4; Fig. 4a), 126 classes (Table S5), 271 orders (Table S6), 401 families (Table S7), 642 genera (Table S8; Fig. 4b) and 289 species (Table S9).

At the phylum level (Fig. 4a), Proteobacteria was the most predominant bacterial phylum in almost all samples (except Virgin-♀-Gut, Firmicutes), with virgin samples showing relatively lower abundance than mated samples within the male RS or female gut, while in female RS, Repeated-♀-RS showed the highest abundance, followed by Virgin-♀-RS and then Multiple-♀-RS. The second and third dominant phyla in most samples were Firmicutes and Bacteroidota (Fig. 4a). In contrast to Proteobacteria, the abundance of Firmicutes was relatively higher in virgin samples than in mated samples in the male RS or female gut, while in female RS, Multiple-♀-RS showed the highest abundance, followed by Repeated-♀-RS and then Virgin-♀-RS.

At the genus level (Fig. 4b), *Enterococcus* was the first dominant bacterial genus

in Virgin-♀-Gut (42.72%), Multiple-♀-RS (26.75%) and Repeated-♂-RS (22.94%); *Asaia* was the first dominant genus in Multiple-♀-Gut (22.21%), Repeated-♀-RS (20.54%), Multiple-♂-RS (17.02%) and Virgin-♀-RS (12.82%); *Vagococcus* (21.68%) and *Providencia* (14.48%) were the first dominant genera of Virgin-♂-RS and Repeated-♀-Gut, respectively. Similar change patterns were observed in *Enterococcus* and *Asaia* within male or female RS, where they had a lower abundance in virgin samples but higher abundance in mated samples. Within the female gut, *Asaia* also showed a lower abundance in virgin samples but a higher abundance in mated samples; however, *Enterococcus* showed a much higher abundance in virgin samples but a lower abundance in mated samples.

LEfSe also showed remarkable differences in the number and taxa of microbial biomarkers between different samples (Fig. S4). The number of biomarkers ranged from 1 to 15 in different samples, with Repeated-♀-Gut, Multiple-♀-RS, Repeated-♀-RS and Virgin-♀-RS being ⩾ 10 while others being ⩽ 6; the taxa of microbial biomarkers were completely different between different samples.

Functional prediction using FAPROTAX

FAPROTAX analysis (Fig. 5a; Fig. S5) showed that chemoheterotrophy (occupying 14.27% to 30.06% of the total annotated functions) and fermentation (10.92% to 25.58%) were the most dominant functions in all samples, with Virgin-♀-Gut showing the highest abundance in both functions. Mating also showed a remarkable effect on these two functions, where mating reduced their abundance in the male RS and female gut while increasing their abundance in the female RS.

As four of the top ten functions were related to human pathogens (Fig. 5a), we further grouped these functions and illustrated them in detail in Figure 5b. the results (Fig. 5b) showed that mating largely increased the abundance of these functions in the

female gut. In male RS, mating decreased them in Repeated-♂-RS but largely increased them in Multiple-♂-RS; while in female RS, mating increased them in Repeated-♀-RS but decreased them in Multiple-♀-RS.

Pathogen and healthy bacterial profiles

Based on the above taxonomy and functional prediction, the possible pathogens and healthy bacteria were further evaluated based on evidence from published studies. A total of 34 animal pathogens were found, including 3 pathogens of insects and others of other animals (Table 2). Statistical analysis showed that mating largely increased the abundance of these possible pathogens in the female gut (Fig. 6a), which was similar to the results of the functional test (Fig. 5b), while in RS, mated males or females might have decreased or increased the abundance of these pathogens (Fig. 6a).

Thirteen *Lactobacillus* spp. (healthy bacteria) were found in *S. frugiperda*, with *Lactobacillus crustorum* and *Lactobacillus murinus* often being the most dominant species in the samples (Table 2). Statistical analysis revealed a higher *Lactobacillus* abundance in the RS of virgin females but lower abundance in the RS of mated females (Fig. 6b). In contrast, *Lactobacillus* abundance was low in the RS of virgin males and increased markedly in the RS of multiple mated males (Fig. 6b).

Discussion

Alpha diversity indices (Table 1; Fig. 3a) and beta diversity analysis (Fig. 3b), as well as LEfSe analysis revealed the variation in bacterial diversity between different samples, and showed that mating induced significant changes in the abundance and composition of the microbiota both in the RS and gut. The microbial composition and abundance in female reproductive organs is likely to be affected by the sexual

transmission of microbes via male genitalia and the ejaculate and by microbes transferred from the cuticle to the reproductive organs during and after mating [57, 58]. A recent study in *C. lectularius* found that mating increased the similarity of the communities of male and female organs and mated individuals harboured bacteria that were found in nonmated individuals of the opposite sex but not in nonmated individuals of the same sex, suggesting that bacteria are sexually transmitted [25]. In the present study, we also found that the RS of mated individuals harboured OTUs that were found in virgin individuals of the opposite sex but not in virgin individuals of the same sex (Fig. 2a-d). However, we did not find that mating increased the similarity of the communities of male and female RS (Fig. S3a-c), which might be because mating differentially affected the microbiota diversity of male and female RS (Table 1). The vaginal microbiota may come from the gut or through a mother-to-child transfer [3], and the vaginal microbiota or invading bacteria may enter the haemolymph and other organs via copulatory wounds and the reproductive duct [18, 57, 58]. In the present study, Venn diagrams (Fig. 2e-h) also indicated that the gut of mated individuals harboured OTUs that were found in the RS of virgin individuals but not in the gut of virgin individuals, and the RS of mated individuals harboured OTUs that were found in the gut of virgin individuals but not in the RS of virgin individuals. These studies may partially suggest that mating induced microbiota transmission between male and female RS and between gut and RS. However, more direct evidence is needed.

The obtained 4315 OTUs were classified into 61 phyla (Table S4) and 642 genera (Table S8). Proteobacteria, Firmicutes and Bacteroidota are the top three dominant phyla either in male RS or female RS and gut in *S. frugiperda* (Fig. 4a), which is similar to the results obtained for the female abdomen of the same species [45]. Studies in other insect species also found that Proteobacteria was the predominant phylum in

gut or reproductive organs, such as in the desert locust *Schistocerca gregaria* [59], the citrus fruit fly *Bactrocera minax* [26] and the sawfly *Cephalcia chuxiongica* [60]. Proteobacteria species may play important roles in insects, such as in helping insects fix nitrogen and preventing the proliferation and establishment of pathogens [3, 61-63]. At the genus level, however, the predominant genus is different in different samples; for example, *Enterococcus* is the most dominant genus in Virgin-♀-Gut, Multiple-♀-RS and Repeated-♂-RS, while *Asaia* is the most dominant genus in Multiple-♀-Gut, Repeated-♀-RS, Multiple-♂-RS and Virgin-♀-RS (Fig. 4b). These results further suggest that mating induces significant changes in the composition of the microbiota in both the RS and gut of *S. frugiperda*.

A previous study of the whole abdomen of female *S. frugiperda* showed that mating increased the abundance of pathogens or pathogenic functions [64]. In the present study, we further showed that matings dramatically increased the abundance of pathogens or pathogenic functions in the gut, while in the RS, the change pattern was not always consistent (Fig. 5; Fig. 6a). It is not clear why the abundance of pathogens increased in the gut of mated females. One possible reason for this phenomenon may be the trade-offs between reproduction and immunity, with enhanced reproductive activity limiting immune defence [28]. In this study, we paired males and females for mating after eclosion and collected samples 6 d after eclosion. Mated females usually lay most (approximately 70%) of their egg load at 6 d of age [48]. From the whole-body perspective, mating seems to have reduced the immune activity in *S. litura* [65]. The inconsistent change patterns in female RS may be because mating increased regional immune activity due to male-transferred foreign materials and infections [30, 31, 66, 67].

Taxonomy assignment revealed thirteen *Lactobacillus* spp. in *S. frugiperda*, with

Lactobacillus crustorum and *Lactobacillus murinus* presenting a high abundance in most samples (Fig. 6b). Moreover, we found that the *Lactobacillus* spp. showed a higher abundance in the RS of virgin females and a lower abundance in the RS of virgin males and the gut of virgin females (Fig. 6b). However, mating reduced the abundance of *Lactobacillus* spp. in the RS of females, while their abundance increased in the RS of males, especially in males mated with multiple females (Fig. 6b). More importantly, the RS of virgin females and the RS of multiple mated males were very similar in terms of the composition and abundance of different *Lactobacillus* species, with *Lactobacillus crustorum* showing much higher abundance in both the RS of virgin females and the RS of males mated with multiple females (Fig. 6b). The reverse change patterns of these species in male and female RS suggest that these bacterial strains are sexually transmitted between sexes in *S. frugiperda*.

Lactobacillus spp. are Gram-positive bacilli, which are believed to originate from the gut [68]. Consistently, *Lactobacilli* can also be found in the gut of female *S. frugiperda* (Fig. 6b). In humans, *Lactobacilli* are dominant vaginal microbiota but also are abundant in the gut [69]. *Lactobacilli* in the gut may play multiple functions, such as energy metabolism and physiological and immunologic homeostasis [64, 69]. In the vagina, *Lactobacilli* produced lactate plays an important role in maintaining a low vaginal pH (3.5-4.5), which is crucial to prevent the colonization and development of opportunistic pathogens including those that may translocate from the gut or from males [68, 70, 71]. A number of *Lactobacillus* strains, such as *L. rhamnosus* GR-1, *L. reuteri* RC-14, *L. brevis* CD2 and *L. salivarius* FV2, have been used orally or intravaginally in the treatment of bacterial vaginosis [72, 73]. Three species found in *S. frugiperda*, namely *L. reuteri*, *L. plantarum* and *L. brevis* (Table 2), have also been identified as human ‘healthy’ vaginal bacterial species [3]. However, whether these

Lactobacillus spp. also benefit female moths is still unknown and thus warrants further study. Future studieson the microbiome and metabolite profile interactions and comparative analysis across different taxa from the perspective of evolution and ecosystems are expected to provide deeper insights in this field.

Conclusion

Mating resulted in significant changes in the abundance and composition of the microbiome in the RS and gut of *S. frugiperda*. Bacterial OTUs were classified into 61 phyla and 642 genera, with Proteobacteria, Firmicutes and Bacteroidota being the most predominant phyla either in male RS or female RS and gut. *Enterococcus* and *Asaia* were the dominant genera in most samples. Mating increased the abundance of pathogens or pathogenic functions in the gut, whereas the change range was trivial in the RS. A total of thirteen *Lactobacillus* spp. were found in *S. frugiperda*, with *Lactobacillus crustorum* and *Lactobacillus murinus* showing high abundance. Three *Lactobacillus* species found in *S. frugiperda*, *L. reuteri*, *L. plantarum* and *L. brevis*, have also been identified as the human "healthy" vaginal microbiome. *Lactobacillus* spp. showed higher abundance in the RS of virgin females and lower abundance in the RS of virgin males and the gut of virgin females. Mating reduced their abundance in the RS of females, but increased their abundance in the RS of males, especially in males mated with multiple females. The abundance and composition of *Lactobacillus* species in the RS of multiple mated males are very similar to those of the RS of virgin females, suggesting that these bacteria are transferred from female RS to male RS through mating. The higher abundance of *Lactobacillus* spp. in the RS of female moths and the similarity of *Lactobacillus* species in *S. frugiperda* with human ‘healthy’ vaginal *Lactobacillus* spp. suggest that these bacterial strains may also play a similar

role in the reproductive tract of female moths.

Ethical approval and consent to participate

Not applicable.

Consent for publication

Not applicable.

Availability of data and materials

The raw reads from 16S rDNA gene sequencing were deposited into the NCBI (https://www.ncbi.nlm.nih.gov/) Sequence Read Archive (SRA) database, the login number is PRJNA844607. Other data generated or analysed during this study are included in this article and its Supplementary Information files.

Competing interests

The authors declare that they have no conflicts of interest.

Funding

This study was jointly supported by projects from Science and Technology Planning Project in Key Areas of Yunnan Province (202001BB050002), Basic Research Projects of Yunnan Province (202201AS070025), Joint Special Project of Yunnan Province for Agricultural Basic Research (2018FG001-002) and National Natural Science Foundation Program of P.R. China (31760635).

Authors' contributions

Q.-Y.Z., L.-Y.Z. and P.C. collected the insects; Q.-Y.Z., L.-Y.Z., D.-Y.F., J.X. and H.Y. designed the study; Q.-Y.Z., L.-Y.Z. and D.-Y.F. dissected the insects and extracted DNA; Q.-Y.Z., L.-Y.Z., D.-Y.F. and J.X. analyzed the data; Q.-Y.Z., L.-Y.Z., J.X. and P.C. wrote the paper. All authors read and approved the final manuscript.

Acknowledgements

We thank Prof. Tian-Hua Zhang from Dongchuan Plant Protection Station for his help during insect cellection and Dr. Xu Gu from Yunnan Academy of Biodiversity for her generous assistance during experiments. We also thank Beijin Novogene Bioinformatics Technology Co., Ltd., for assistance during the bioinformatics analysis of the data.

Authors' information

[1]Yunnan Academy of Biodiversity, Southwest Forestry University, Bailong Road 300#, Kunming 650224, China. [2]Key Laboratory for Forest Resources Conservation and Utilization in the Southwest Mountains of China, Ministry of Education, Southwest Forestry University, Bailong Road 300#, Kunming 650224, China. [3]Yunnan Academy of Forestry and Grassland, Lanan Road 2#, Kunming 650201, China. [4]School of Ecology and Environment, Yunnan University, Cuihu North Road 2#, Kunming 650091, China.

References

1. Voirol LRP, Frago E, Kaltenpoth M, Hilker M, Fatouros NE. Bacterial symbionts in Lepidoptera: Their diversity, transmission, and impact on the host. Front Microbiol. 2018;9:556; doi: 10.3389/fmicb.2018.00556.

2. Bar-Shmuel N, Behar A, Segoli M. What do we know about biological nitrogen fixation in insects? Evidence and implications for the insect and the ecosystem. Insect Sci. 2020;27(3):392-403; doi: 10.1111/1744-7917.12697.
3. Amabebe E, Anumba DOC. Female gut and genital tract microbiota-induced crosstalk and differential effects of short-chain fatty acids on immune sequelae. Front Immunol. 2020;11:2184; doi: 10.3389/fimmu.2020.02184.
4. He Z, Ma Y, Yang S, Zhang S, Liu S, Xiao J, et al. Gut microbiota-derived ursodeoxycholic acid from neonatal dairy calves improves intestinal homeostasis and colitis to attenuate extended-spectrum β-lactamase-producing enteroaggregative *Escherichia coli* infection. Microbiome. 2022;10:79; doi: 10.1186/s40168-022-01269-0.
5. Xu F, Fu Y, Sun T-y, Jiang Z, Miao Z, Shuai M, et al. The interplay between host genetics and the gut microbiome reveals common and distinct microbiome features for complex human diseases. Microbiome. 2020;8(1):145; doi: 10.1186/s40168-020-00923-9.
6. Tayasu I, Sugimoto A, Wada E, Abe T. Xylophagous termites depending on atmospheric nitrogen. Naturwissenschaften. 1994;81(5):229-31; doi: 10.1007/s001140050063.
7. Florez LV, Biedermann PHW, Engl T, Kaltenpoth M. Defensive symbioses of animals with prokaryotic and eukaryotic microorganisms. Nat Prod Rep. 2015;32(7):904-36; doi: 10.1039/c5np00010f.
8. van den Bosch TJM, Welte CU. Detoxifying symbionts in agriculturally important pest insects. Microb Biotechnol. 2017;10(3):531-40; doi: 10.1111/1751-7915.12483.
9. Rowe M, Veerus L, Trosvik P, Buckling A, Pizzari T. The reproductive microbiome: an emerging driver of sexual selection, sexual conflict, mating systems, and reproductive isolation. Trends Ecol Evol. 2021;36(1):98-; doi: 10.1016/j.tree.2020.10.015.
10. Schoenmakers S, Steegers-Theunissen R, Faas M. The matter of the reproductive microbiome. Obst Med. 2019;12(3):107-15; doi: 10.1177/1753495x18775899.
11. Fraczek M, Szumala-Kakol A, Dworacki G, Sanocka D, Kurpisz M. In vitro reconstruction of inflammatory reaction in human semen: effect on sperm DNA fragmentation. J Reprod Immunol. 2013;100(1, Sp. Iss. SI):76-85; doi: 10.1016/j.jri.2013.09.005.
12. Lockhart AB, Thrall PH, Antonovics J. Sexually transmitted diseases in animals: ecological and evolutionary implications. Biol Rev. 1996;71(3):415-71; doi: 10.1111/j.1469-185X.1996.tb01281.x.
13. Chen C, Song X, Wei W, Zhong H, Dai J, Lan Z, et al. The microbiota continuum along the female reproductive tract and its relation to uterine-related diseases. Nat Commun. 2017;8:875; doi: 10.1038/s41467-017-00901-0.
14. Ravel J, Gajer P, Abdo Z, Schneider GM, Koenig SSK, McCulle SL, et al. Vaginal microbiome of reproductive-age women. P Natl Acad Sci USA. 2011;108(Suppl. 1):4680-7; doi: 10.1073/pnas.1002611107.
15. Marconi C, El-Zein M, Ravel J, Ma B, Lima MD, Carvalho NS, et al. Characterization of the vaginal microbiome in women of reproductive age from 5 regions in Brazil. Sex Transm Dis. 2020;47(8):562-9; doi: 10.1097/olq.0000000000001204.
16. Younes JA, Lievens E, Hummelen R, van der Westen R, Reid G, Petrova MI. Women and their microbes: the unexpected friendship. Trends Microbiol. 2018;26(1):16-32; doi: 10.1016/j.tim.2017.07.008.

17. Tachedjian G, Aldunate M, Bradshaw CS, Cone RA. The role of lactic acid production by probiotic *Lactobacillus* species in vaginal health. Res Microbiol. 2017;168(9-10, Sp. Iss. SI):782-92; doi: 10.1016/j.resmic.2017.04.001.
18. Bellinvia S, Johnston PR, Mbedi S, Otti O. Mating changes the genital microbiome in both sexes of the common bedbug *Cimex lectularius* across populations. Proc R Soc Lond B Biol Sci. 2020;287(1926):20200302; doi: 10.1098/rspb.2020.0302.
19. Otti O, McTighe AP, Reinhardt K. In vitro antimicrobial sperm protection by an ejaculate-like substance. Funct Ecol. 2013;27(1):219-26; doi: 10.1111/1365-2435.12025.
20. Akami M, Ren XM, Qi XW, Mansour A, Gao BL, Cao S, et al. Symbiotic bacteria motivate the foraging decision and promote fecundity and survival of *Bactrocera dorsalis* (Diptera: Tephritidae). BMC Microbiol. 2019;19(1):229; doi: 10.1186/s12866-019-1607-3.
21. Noman MS, Shi G, Liu L-J, Li Z-H. Diversity of bacteria in different life stages and their impact on the development and reproduction of *Zeugodacus tau* (Diptera: Tephritidae). Insect Sci. 2021;28(2):363-76; doi: 10.1111/1744-7917.12768.
22. El Aila NA, Tency I, Claeys G, Verstraelen H, Saerens B, Santiago GLDS, et al. Identification and genotyping of bacteria from paired vaginal and rectal samples from pregnant women indicates similarity between vaginal and rectal microflora. BMC Infec Dis. 2009;9:167-; doi: 10.1186/1471-2334-9-167.
23. El Aila NA, Tency I, Saerens B, De Backer E, Cools P, Santiago GLdS, et al. Strong correspondence in bacterial loads between the vagina and rectum of pregnant women. Res Microbiol. 2011;162(5):506-13; doi: 10.1016/j.resmic.2011.04.004.
24. Riviere A, Selak M, Lantin D, Leroy F, De Vuyst L. Bifidobacteria and butyrate-producing colon bacteria: Importance and strategies for their stimulation in the human gut. Front Microbiol. 2016;7:979; doi: 10.3389/fmicb.2016.00979.
25. Bellinvia S, Johnston PR, Reinhardt K, Otti O. Bacterial communities of the reproductive organs of virgin and mated common bedbugs, *Cimex lectularius*. Ecol Entomol. 2020;45(1):142-54; doi: 10.1111/een.12784.
26. Wang A, Yao Z, Zheng W, Zhang H. Bacterial communities in the gut and reproductive organs of *Bactrocera minax* (Diptera: Tephritidae) based on 454 pyrosequencing. Plos One. 2014;9(9):e106988; doi: 10.1371/journal.pone.0106988.
27. Otti O, Deines P, Hammerschmidt K, Reinhardt K. Regular wounding in a natural system: bacteria associated with reproductive organs of bedbugs and their quorum sensing abilities. Front Immunol. 2017;8:1855-; doi: 10.3389/fimmu.2017.01855.
28. Schwenke RA, Lazzaro BP, Wolfner MF. Reproduction-immunity trade-offs in insects. Annu Rev Entomol. 2016;61:239-56; doi: 10.1146/annurev-ento-010715-023924.
29. Wigby S, Suarez SS, Lazzaro BP, Pizzari T, Wolfner MF. Sperm success and immunity. Curr Top Dev Biol. 2019;135:287-313; doi: 10.1016/bs.ctdb.2019.04.002.
30. Okada K, Suzaki Y, Sasaki R, Katsuki M. Fitness costs of polyandry to female cigarette beetle *Lasioderma serricorne*. Behav Ecol Sociobiol. 2017;71:86.
31. Oku K, Price TAR, Wedell N. Does mating negatively affect female immune defences in insects? Anim Biol. 2019;69(1):117-36; doi: 10.1163/15707563-20191082.
32. Delbare SYN, Chow CY, Wolfner MF, Clark AG. Roles of female and male genotype in post-mating responses in *Drosophila melanogaster*. J Hered. 2017;108(7):740-53; doi: 10.1093/jhered/esx081.

33. Ahmed-Braimah YH, Wolfner MF, Clark AG. Differences in postmating transcriptional responses between conspecific and heterospecific matings in *Drosophila*. Mol Biol Evol. 2021;38(3):986-99; doi: 10.1093/molbev/msaa264.
34. Hurst GDD, Sharpe RG, Broomfield AH, Walker LE, Majerus TMO, Zakharov IA, et al. Sexually-transmitted disease in a promiscuous insect, *Adalia bipunctata*. Ecol Entomol. 1995;20(3):230-6.
35. CABI. Invasive species compendium: *Spodoptera frugiperda* (fall armyworm) Datasheet. https://www.cabi.org/isc/datasheet/29810 (2020). Accessed 26 Nov. 2020.
36. Kumar M, Gupta GP, Rajam MV. Silencing of acetylcholinesterase gene of *Helicoverpa armigera* by siRNA affects larval growth and its life cycle. J Insect Physiol. 2009;55(3):273-8; doi: 10.1016/j.jinsphys.2008.12.005.
37. Zhang L, Liu B, Jiang YY, Liu J, Wu KM, Xiao YT. Molecular characterization analysis of fall armyworm populations in China. Plant Prot (China). 2019;45(4):20-7.
38. Westbrook JK, Nagoshi RN, Meagher RL, Fleischer SJ, Jairam S. Modeling seasonal migration of fall armyworm moths. Int J Biometeorol. 2016;60(2):255-67; doi: 10.1007/s00484-015-1022-x.
39. Montezano DG, Specht A, Sosa-Gomez DR, Roque-Specht VF, Sousa-Silva JC, Paula-Moraes SV, et al. Host plants of *Spodoptera frugiperda* (Lepidoptera: Noctuidae) in the Americas. Afr Entomol. 2018;26(2):286-300; doi: 10.4001/003.026.0286.
40. APRD. Arthropod pesticide resistance database. http://www.pesticide-resistance.org/ (2021). Accessed 18 May 2021.
41. Li Y, Zhang S, Wang X, Xie X, Liang P, Zhang L, et al. Current status of insecticide resistance in *Spodoptera frugiperda* and strategies for its chemical control. Plant Prot (China). 2019;45:14-9.
42. Ferrari J, Vavre F. Bacterial symbionts in insects or the story of communities affecting communities. Philos T Roy Soc B. 2011;366(1569):1389-400; doi: 10.1098/rstb.2010.0226.
43. Perilla-Henao LM, Casteel CL. Vector-borne bacterial plant pathogens: interactions with Hemipteran insects and plants. Front Plant Sci. 2016;7:1163; doi: 10.3389/fpls.2016.01163.
44. Beck JJ, Vannette RL. Harnessing insect-microbe chemical communications to control insect pests of agricultural systems. J Agr Food Chem. 2017;65(1):23-8; doi: 10.1021/acs.jafc.6b04298.
45. Zhang L-Y, Yu H, Fu D-Y, Xu J, Yang S, Ye H. Mating leads to a decline in the diversity of symbiotic microbiomes and promiscuity increased pathogen abundance in a moth. Front Microbiol. 2022;13:878856; doi: 10.3389/fmicb.2022.878856.
46. Li W, Zou WJ, Wang LH. The bionomics and control of *Prodenia litura* in Kunming. Southwest China J Agric Sci. 2006;19:85-9.
47. Dong Q-J, Zhou J-C, Zhu K-H, Z-T Z, Dong H. A simple method for identifiying sexuality of *Spodoptera frugiperd* (J. E. Smith) pupae and adults. Plant Prot (China). 2019;45(5):96-8.
48. Zhang L-Y, Wang F, Wan X-S, Xu J, Ye H. Reproductive behavior and circadian rhythms of *Spodoptera frugiperda*. J Environ Entomol. 2022;(in press):https://kns.cnki.net/kcms/detail/44.1640.Q.20211014.1311.002.html.
49. Doyle J, Doyle J. A rapid DNA isolation procedure for small quantities of fresh leaf tissues. Phytochemical Bulletin. 1987;19:11-5.
50. Magoč T, L SS. FLASH: fast length adjustment of short reads to improve genome assemblies. Bioinformatics 2011;27(21):2957-63.

51. Bokulich NA, Subramanian S, Faith JJ, Gevers D, Gordon JI, Knight R, et al. Quality-filtering vastly improves diversity estimates from Illumina amplicon sequencing. Nat Methods. 2013;10(1):57-9.
52. T R, T F, B N, C Q, F M. VSEARCH: a versatile open source tool for metagenomics. PeerJ. 2016;4:e2584.
53. Edgar RC. UPARSE: highly accurate OTU sequences from microbial amplicon reads. Nat Methods. 2013;10(10):996-8; doi: 10.1038/nmeth.2604.
54. Wang Q, Garrity GM, Tiedje JM, Cole JR. Naive Bayesian classifier for rapid assignment of rRNA sequences into the new bacterial taxonomy. Appl Environ Microbal. 2007;73(16):5261-7; doi: 10.1128/aem.00062-07.
55. Louca S, Parfrey LW, Doebeli M. Decoupling function and taxonomy in the global ocean microbiome. Science. 2016;353(6305):1272-7; doi: 10.1126/science.aaf4507.
56. Djemiel C, Maron P-A, Terrat S, Dequiedt S, Cottin A, Ranjard L. Inferring microbiota functions from taxonomic genes: a review. GigaScience. 2022;11(1):giab090; doi: 10.1093/gigascience/giab090.
57. Lange R, Reinhardt K, Michiels NK, Anthes N. Functions, diversity, and evolution of traumatic mating. Biol Rev. 2013;88(3):585-601; doi: 10.1111/brv.12018.
58. Reinhardt K, Anthes N, Lange R. Copulatory wounding and traumatic insemination. Cold Spring Perspec Biol. 2015;7(5):a017582; doi: 10.1101/cshperspect.a017582.
59. Dillon RJ, Webster G, Weightman AJ, Charnley AK. Diversity of gut microbiota increases with aging and starvation in the desert locust. Anton Leeuw. 2010;97(1):69-77; doi: 10.1007/s10482-009-9389-5.
60. Yu H, Du C-M, Shi M-R, Feng L, Fu D-Y, Xu J, et al. The diversity and function of intestinal microorganisms in four geographic *Cephalcia chuxiongica* (a pine defoliator) populations. J Appl Entomol. 2021;145(5):394-405; doi: https://doi.org/10.1111/jen.12858.
61. Behar A, Yuval B, Jurkevitch E. Enterobacteria-mediated nitrogen fixation in natural populations of the fruit fly *Ceratitis capitata*. Mol Ecol. 2005;14(9):2637-43; doi: 10.1111/j.1365-294X.2005.02615.x.
62. Dixon R, Kahn D. Genetic regulation of biological nitrogen fixation. Nat Rev Microbiol. 2004;2(8):621-31; doi: 10.1038/nrmicro954.
63. Dillon RJ, Dillon VM. The gut bacteria of insects: Nonpathogenic interactions. Annu Rev Entomol. 2004;49:71-92; doi: 10.1146/annurev.ento.49.061802.123416.
64. Amabebe E, Robert FO, Agbalalah T, Orubu ESF. Microbial dysbiosis-induced obesity: role of gut microbiota in homoeostasis of energy metabolism. Brit J Nutr. 2020;123(10):1127-37; doi: 10.1017/S0007114520000380.
65. Gao B, Song XQ, Yu H, Fu DY, Xu J, Ye H. Mating-induced differential expression in genes related to reproduction and immunity in *Spodoptera litura* (Lepidoptera: Noctuidae) female moths. J Insect Sci. 2020;20(1):10; doi: 10.1093/jisesa/ieaa003.
66. Whitlow CB. Bacterial sexually transmitted diseases. Clin Colon Rectal Surg. 2004;17(4):209-14; doi: 10.1055/s-2004-836940.
67. Smith G, Dobson AP. Sexually transmitted diseases in animals. Parasitol Today. 1992;8(5):159-66; doi: 10.1016/0169-4758(92)90010-y.
68. Amabebe E, Anumba DOC. The vaginal microenvironment: The physiologic role of Lactobacilli. Front Med. 2018;5:181; doi: 10.3389/fmed.2018.00181.

69. Kobyliak N, Virchenko O, Falalyeyeva T. Pathophysiological role of host microbiota in the development of obesity. Nutrit J. 2016;15:43-; doi: 10.1186/s12937-016-0166-9.
70. Tester R, Al-Ghazzewi FH. Intrinsic and extrinsic carbohydrates in the vagina: A short review on vaginal glycogen. Int J Biol Macromol. 2018;112:203-6; doi: 10.1016/j.ijbiomac.2018.01.166.
71. Ceccarani C, Foschi C, Parolin C, D'Antuono A, Gaspari V, Consolandi C, et al. Diversity of vaginal microbiome and metabolome during genital infections. Sci Rep. 2019;9:14095; doi: 10.1038/s41598-019-50410-x.
72. Ling Z, Liu X, Chen W, Luo Y, Yuan L, Xia Y, et al. The restoration of the vaginal microbiota after treatment for bacterial vaginosis with metronidazole or probiotics. Microbial Ecol. 2013;65(3):773-80; doi: 10.1007/s00248-012-0154-3.
73. Mastromarino P, Vitali B, Mosca L. Bacterial vaginosis: a review on clinical trials with probiotics. New Microbiol. 2013;36(3):229-38.
74. Salas B, Conway HE, Schuenzel EL, Hopperstad K, Vitek C, Vacek DC. *Morganella morganii* (Enterobacteriales: Enterobacteriaceae) is a lethal pathogen of Mexican fruit fly (Diptera: Tephritidae) larvae. Fla Entomol. 2017;100(4):743-51.
75. Maciel-Vergara G, Jensen AB, Eilenberg J. Cannibalism as a possible entry route for opportunistic pathogenic bacteria to insect hosts, exemplified by *Pseudomonas aeruginosa*, a pathogen of the giant mealworm *Zophobas morio*. Insects. 2018;9(3):88; doi: 10.3390/insects9030088.
76. Au CWH, Yap DYH, Chan JFW, Yip TPS, Chan TM. Exit site infection and peritonitis due to *Serratia species* in patients receiving peritoneal dialysis: Epidemiology and clinical outcomes. Nephrology. 2021;26(3):255-61; doi: 10.1111/nep.13813.
77. Huang C. Extensively drug-resistant *Alcaligenes faecalis* infection. BMC Infec Dis. 2020;20(1):833-; doi: 10.1186/s12879-020-05557-8.
78. Shao S, Guo X, Guo P, Cui Y, Chen Y. Roseomonas mucosa infective endocarditis in patient with systemic lupus erythematosus: case report and review of literature. BMC Infec Dis. 2019;19(1):140-; doi: 10.1186/s12879-019-3774-0.
79. Flannagan RS. Burkholderia cenocepacia infection: disruption of phagocyte immune functions through Rho GTPase inactivation. Cell Adhes Migr. 2012;6(4):297-301; doi: 10.4161/cam.20487.
80. Dragan AL, Voth DE. *Coxiella burnetii*: international pathogen of mystery. Microb Infect. 2020;22(3):100-10; doi: 10.1016/j.micinf.2019.09.001.
81. Yuan F, Huang Z, Yang T, Wang G, Li P, Yang B, et al. Pathogenesis of *Proteus mirabilis* in catheter-associated urinary tract infections. Urol Int. 2021;105(5-6):354-61; doi: 10.1159/000514097.
82. Coker C, Poore CA, Li X, Mobley HLT. Pathogenesis of *Proteus mirabilis* urinary tract infection. Microb Infect. 2000;2(12):1497-505; doi: 10.1016/s1286-4579(00)01304-6.
83. Adegoke AA, Stenstrom TA, Okoh AI. *Stenotrophomonas maltophilia* as an emerging ubiquitous pathogen: looking beyond contemporary antibiotic therapy. Front Microbiol. 2017;8:2276; doi: 10.3389/fmicb.2017.02276.
84. Huang Y-T, Chen J-M, Ho B-C, Wu Z-Y, Kuo RC, Liu P-Y. Genome sequencing and comparative analysis of *Stenotrophomonas acidaminiphila* reveal evolutionary insights into sulfamethoxazole resistance. Front Microbiol. 2018;9:e1013; doi: 10.3339/fmicb.2018.01013.

85. Dignani MC, Grazziutti M, Anaissie E. *Stenotrophomonas maltophilia* infections. Sem Resp Crit Care M. 2003;24(1):89-98; doi: 10.1055/s-2003-37920.
86. Kozińska A, Paździor E, Pękala A, Niemczuk W. *Acinetobacter johnsonii* and *Acinetobacter lwoffii* - the emerging fish pathogens. B Veter I Pulawy. 2014;58:193-9.
87. Peleg AY, Seifert H, Paterson DL. *Acinetobacter baumannii*: Emergence of a successful pathogen. Clin Microbiol Rev. 2008;21(3):538-82; doi: 10.1128/cmr.00058-07.
88. Chen T-L, Siu L-K, Lee Y-T, Chen C-P, Huang L-Y, Wu RC-C, et al. *Acinetobacter baylyi* as a pathogen for opportunistic infection. J Clin Microbiol. 2008;46(9):2938-44; doi: 10.1128/jcm.00232-08.
89. Loubinoux J, Mihaila-Amrouche L, Le Fleche A, Pigne E, Huchon G, Grimont PAD, et al. Bacteremia caused by *Acinetobacter ursingii*. J Clin Microbiol. 2003;41(3):1337-8; doi: 10.1128/JCM.41.3.1337-1338.2003.
90. Li J, Cao JL, Wang X, Liu N, Wang WM, Luo Y. *Acinetobacter pittii*, an emerging new multi-drug resistant fish pathogen isolated from diseased blunt snout bream (*Megalobrama amblycephala* Yih) in China. Appl Microbiol Biot. 2017;101(16):6459-71; doi: 10.1007/s00253-017-8392-4.
91. de Amorim AM, Nascimento JDS. Acinetobacter: an underrated foodborne pathogen? J Infec Devel Countries. 2017;11(2):111-4; doi: 10.3855/j.idc.8418.
92. Passalacqua KD, Bergman NH. *Bocillus anthracis*: interactions with the host and establishment of inhalational anthrax. Future Microbiol. 2006;1(4):397-415; doi: 10.2217/17460913.1.4.397.
93. Tao Z, Zhang L, Zhang QQ, Lv T, Chen R, Wang LJ, et al. The pathogenesis of *Streptococcus anginosus* in aerobic vaginitis. Infec Drug Resis. 2019;12:3745-54; doi: 10.2147/idr.S227883.
94. Martin T, Aziz H. *Bacteroides fragilis*: a case study of bacteremia and septic arthritis. Clinic Lab Sci. 2009;22(3):131-5.
95. Bokhari S, Abbas N, Singh M, Cindrich RB, Zeana C. *Empedobacter brevis* bacteremia in a patient infected with HIV: Case report and review of literature. Case Rep Infect Dis. 2015;2015:813528; doi: 10.1155/2015/813528.
96. Licker M, Sorescu T, Rus M, Cirlea N, Horhat F, Jurescu C, et al. Extensively drug-resistant *Myroides odoratimimus* - a case series of urinary tract infections in immunocompromised patients. Infec Drug Resis. 2018;11:743-9; doi: 10.2147/idr.S161069.

Table 1 Alpha diversity indices of bacteria in different samples of *S. frugiperda*

Sample name	Number of OTUs	Observed species	Shannon	Simpson	Chao1	Cood's coverage
Virgin-♂-RS	1188	488±87	3.760±0.151	0.8542±0.0187	564.85±105.72	0.9978±0.00048
Repeated-♂-RS	2091	925±98	4.366±0.246	0.8813±0.0123	1038.29±100.42	0.9965±0.00029
Multiple-♂-RS	1047	409±90	3.752±0.348	0.8525±0.0290	465.50±106.31	0.999±0.00050
Virgin-♀-RS	2057	753±115	4.505±0.132	0.8505±0.0213	818.33±120.07	0.997±0.00065
Repeated-♀-RS	1094	380±117	3.101±0.172	0.7742±0.0271	446.22±129.61	0.998±0.00048
Multiple-♀-RS	1597	531±139	4.099±0.514	0.8622±0.0419	596.30±158.78	0.998±0.00063
Virgin-♀-Gut	1215	564±65	3.512±0.357	0.7708±0.0419	628.05±70.90	0.998±0
Repeated-♀-Gut	2041	845±125	4.649±0.297	0.8740±0.0195	937.89±150.30	0.997±0.00095
Multiple-♀-Gut	1283	471±79	3.442±0.200	0.8020±0.0394	544.62±92.29	0.998±0.00041

Table 2 Possible pathogens and healthy vaginal bacteria profiles in *S. frugiperda*

Functional Group	Taxonomy			Functional Annotation	Reference
	Phylum	Order	Species		
Pathogen	Proteobacteria	Enterobacterales	*Morganella morganii*	Insect pathogen	[74]
	Proteobacteria	Enterobacterales	*Serratia marcescens*	Insect pathogen	[1, 75]
	Proteobacteria	Pseudomonadales	*Pseudomonas aeruginosa*	Insect pathogen	[75]
	Proteobacteria	Enterobacterales	*Serratia* spp. (OTU_1130; OTU_3075)	Animal pathogen	[1, 76]
	Proteobacteria	Acetobacterales	*Alcaligenes faecalis*	Human pathogen	[77]
	Proteobacteria	Acetobacterales	*Roseomonas* spp. (OTU_2563; OTU_3173; OTU_3212)	Human pathogen	[78]
	Proteobacteria	Burkholderiales	*Burkholderia cenocepacia*	Human pathogen	[79]
	Proteobacteria	Coxiellales	*Coxiella* spp. (OTU_2646; OTU_3289; OTU_3667; OTU_4069)	Human pathogen	[80]
	Proteobacteria	Enterobacterales	*Proteus mirabilis*	Human pathogen	[81, 82]
	Proteobacteria	Xanthomonadales	*Stenotrophomonas maltophilia*	Human pathogen	[83]
	Proteobacteria	Xanthomonadales	*Stenotrophomonas acidaminiphila*	Potential human pathogen	[84]
	Proteobacteria	Xanthomonadales	*Stenotrophomonas* spp. (OTU_3295)	Potential human pathogen	[85]
	Proteobacteria	Pseudomonadales	*Acinetobacter lwoffii*	Human and fish pathogen	[86, 87]
	Proteobacteria	Pseudomonadales	*Acinetobacter baylyi*	Human pathogen	[88]
	Proteobacteria	Pseudomonadales	*Acinetobacter ursingii*	Human pathogen	[89]
	Proteobacteria	Pseudomonadales	*Acinetobacter pittii*	Human pathogen	[90]
	Proteobacteria	Pseudomonadales	*Acinetobacter* spp. (OUT_11; OTU_1250; OTU_2772; OTU_2973; OTU_3124; OTU_3319; OTU_4025)	Human pathogen	[91]
	Firmicutes	Bacillales	*Bacillus anthracis*	Human pathogen	[92]
	Firmicutes	Lactobacillales	*Streptococcus anginosus*	Human pathogen	[93]
	Bacteroidota	Bacteroidales	*Bacteroides fragilis*	Human pathogen	[94]
	Bacteroidota	Flavobacteriales	*Empedobacter brevis*	Human pathogen	[95]
	Bacteroidota	Flavobacteriales	*Myroides odoratimimus*	Human pathogen	[96]
Healthy vaginal bacteria	Firmicutes	Lactobacillales	*Lactobacillus crustorum*	Healthy vaginal microbiome	[9, 10]
	Firmicutes	Lactobacillales	*Lactobacillus reuteri*	Healthy vaginal microbiome	[9, 10]
	Firmicutes	Lactobacillales	*Lactobacillus curvatus*	Healthy vaginal microbiome	[9, 10]
	Firmicutes	Lactobacillales	*Lactobacillus plantarum*	Healthy vaginal microbiome	[9, 10]
	Firmicutes	Lactobacillales	*Lactobacillus ruminis*	Healthy vaginal microbiome	[9, 10]
	Firmicutes	Lactobacillales	*Lactobacillus brevis*	Healthy vaginal microbiome	[9, 10]
	Firmicutes	Lactobacillales	*Lactobacillus murinus*	Healthy vaginal microbiome	[9, 10]
	Firmicutes	Lactobacillales	*Lactobacillus rossiae*	Healthy vaginal microbiome	[9, 10]
	Firmicutes	Lactobacillales	*Lactobacillus* spp. (OTU_3161; OTU_3392; OTU_4098; OTU_89; OTU_118)	Healthy vaginal microbiome	[9, 10]

Figure Captions

Fig. 1 Experimental design and sample information of *S. frugiperda*. (a) Treatment and sampling method and sample name; (b) A male and a female mating in *S. frugiperda*; (c) Sampled male reproductive system, in which ae = Aedeagus, ag = Accessory gland, pg = Paired gland, t = Testis, ug = Unpaired gland, vd = Vas deferens, and vs = Vesicula seminalis; (d) Sampled female reproductive system, in which a = Accessory gland reservoir, bc = Bursa copulatrix, ds = Ductus seminalis, ov = Ovary, sc = Spermatheca, sd = Spermathecal duct, sg = Spermathecal gland, vlv = Vulva, and vs = Vestibulum; and (e) Sampled female gut.

Fig. 2 The OTU Venn diagrams of different samples from *S. frugiperda*. (a) Virgin-♂-RS vs. Virgin-♀-RS vs. Repeated-♀-RS; (b) Virgin-♂-RS vs. Virgin-♀-RS vs. Multiple-♀-RS; (c) Virgin-♂-RS vs. Virgin-♀-RS vs. Repeated-♂-RS; (d) Virgin-♂-RS vs. Virgin-♀-RS vs. Multiple-♂-RS; (e) Virgin-♀-Gut vs. Virgin-♀-RS vs. Repeated-♀-Gut; (f) Virgin-♀-Gut vs. Virgin-♀-RS vs. Multiple-♀-Gut; (g) Virgin-♀-Gut vs. Virgin-♀-RS vs. Repeated-♀-RS; and (h) Virgin-♀-Gut vs. Virgin-♀-RS vs. Multiple-♀-RS.

Fig. 3 Shannon diversity indices (a) and PCoA ordination based on Bray–Curtis distances (b) of different samples from *S. frugiperda*. In subgraph (a), bars with different letters are significantly different ($P < 0.05$).

Fig. 4 Taxonomic assignment of bacterial OTUs at the phylum (top 10; a) and genus (top 10; b) levels in different samples of *S. frugiperda*.

Fig. 5 The relative abundance of dominant bacterial functional groups (top 10) (a) and the relative abundance of different pathogenic functions (b) of different samples from *S. frugiperda* (predicted by FAPROTAX).

Fig. 6 The relative abundance of possible pathogens (a) and healthy vaginal bacteria (b) in different samples of *S. frugiperda*.

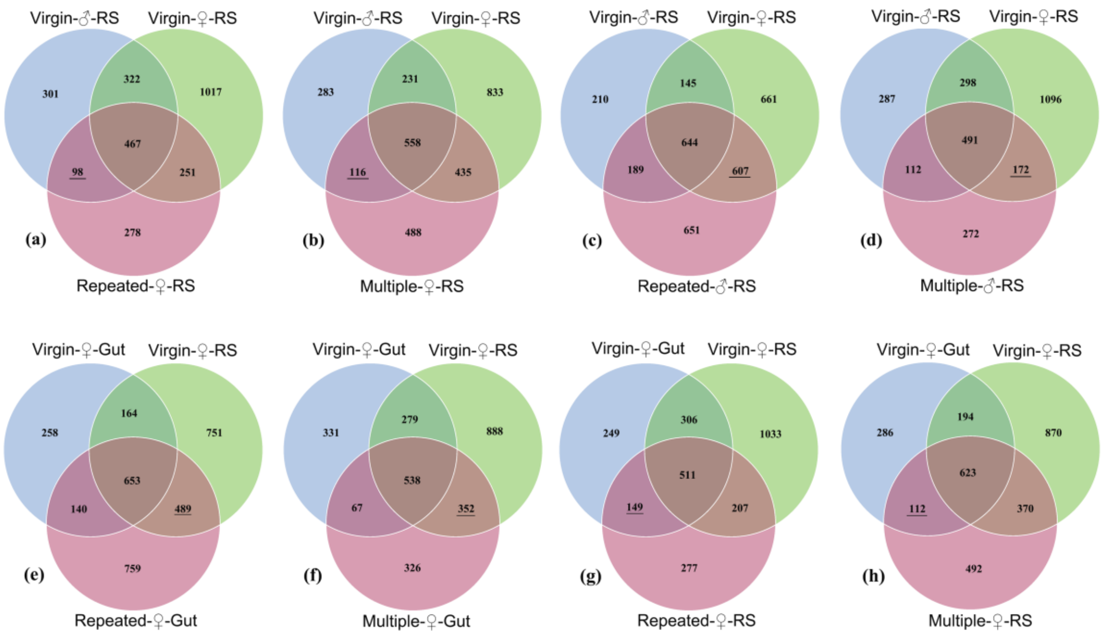

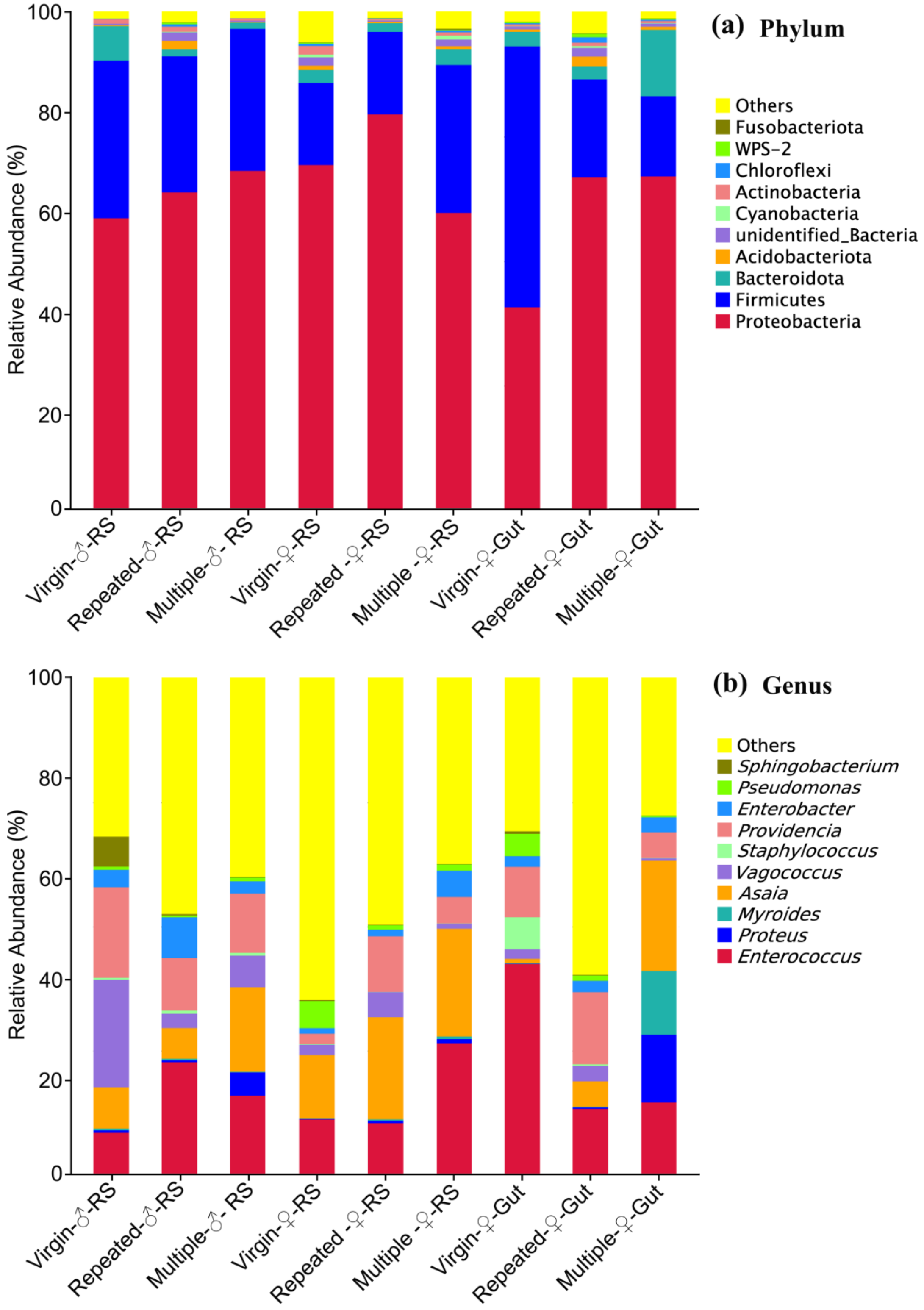
(a) Phylum
Relative Abundance (%)
100
80
60
40
20
0
Others
Fusobacteriota
WPS−2
Chloroflexi
Actinobacteria
Cyanobacteria
unidentified_Bacteria
Acidobacteriota
Bacteroidota
Firmicutes
Proteobacteria
Virgin-♂-RS
Repeated-♂-RS
Multiple-♂- RS
Virgin-♀-RS
Repeated -♀-RS
Multiple -♀-RS
Virgin-♀-Gut
Repeated-♀-Gut
Multiple-♀-Gut
(b) Genus
Relative Abundance (%)
100
80
60
40
20
0
Others
Sphingobacterium
Pseudomonas
Enterobacter
Providencia
Staphylococcus
Vagococcus
Asaia
Myroides
Proteus
Enterococcus
Virgin-♂-RS
Repeated-♂-RS
Multiple-♂- RS
Virgin-♀-RS
Repeated -♀-RS
Multiple -♀-RS
Virgin-♀-Gut
Repeated-♀-Gut
Multiple-♀-Gut

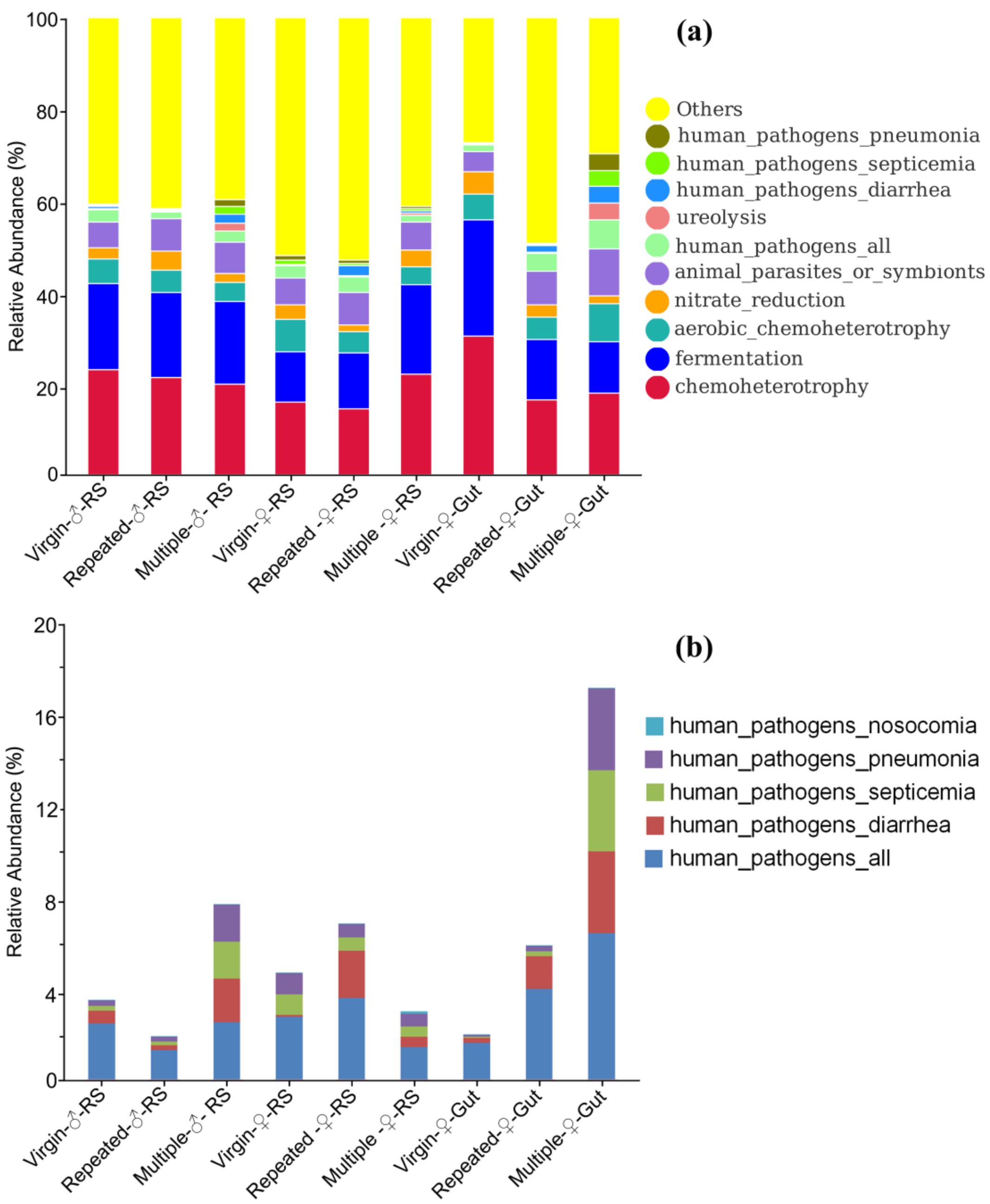
(a)
Relative Abundance (%)
100
80
60
40
20
0
Others
human_pathogens_pneumonia
human_pathogens_septicemia
human_pathogens_diarrhea
ureolysis
human_pathogens_all
animal_parasites_or_symbionts
nitrate_reduction
aerobic_chemoheterotrophy
fermentation
chemoheterotrophy
Virgin-♂-RS
Repeated-♂-RS
Multiple-♂- RS
Virgin-♀-RS
Repeated -♀-RS
Multiple -♀-RS
Virgin-♀-Gut
Repeated-♀-Gut
Multiple-♀-Gut
(b)
Relative Abundance (%)
20
16
12
8
4
0
human_pathogens_nosocomia
human_pathogens_pneumonia
human_pathogens_septicemia
human_pathogens_diarrhea
human_pathogens_all
Virgin-♂-RS
Repeated-♂-RS
Multiple-♂- RS
Virgin-♀-RS
Repeated -♀-RS
Multiple -♀-RS
Virgin-♀-Gut
Repeated-♀-Gut
Multiple-♀-Gut

(a)

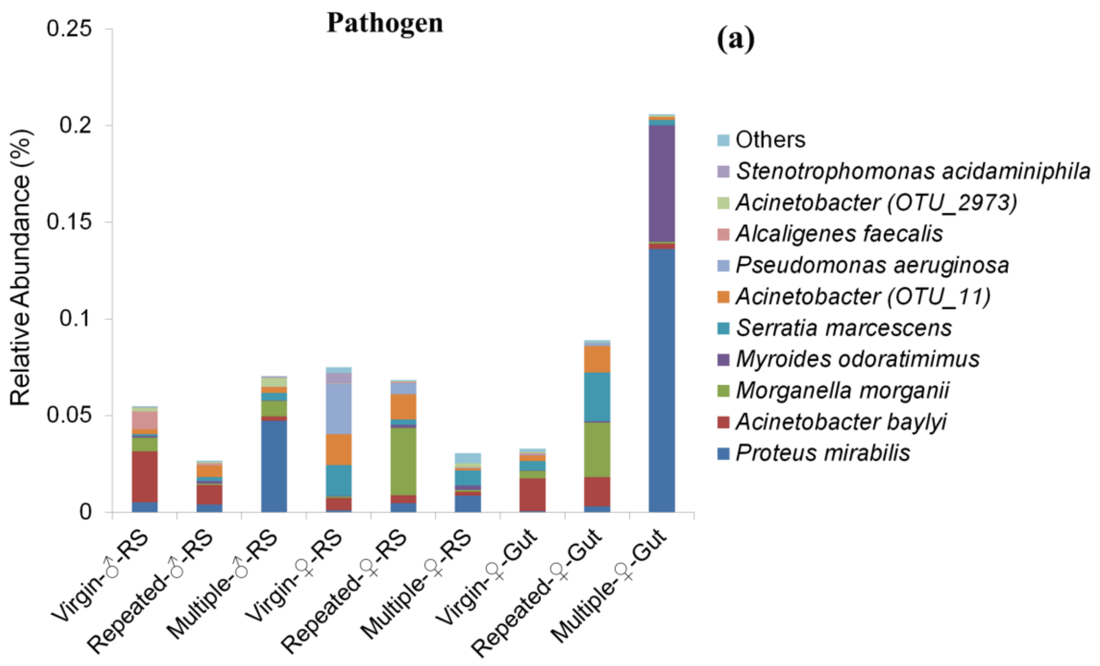
Pathogen
Relative Abundance (%)
0.25
0.2
0.15
0.1
0.05
0
Virgin-♂-RS
Repeated-♂-RS
Multiple-♂-RS
Virgin-♀-RS
Repeated-♀-RS
Multiple-♀-RS
Virgin-♀-Gut
Repeated-♀-Gut
Multiple-♀-Gut
Others
Stenotrophomonas acidaminiphila
Acinetobacter (OTU_2973)
Alcaligenes faecalis
Pseudomonas aeruginosa
Acinetobacter (OTU_11)
Serratia marcescens
Myroides odoratimimus
Morganella morganii
Acinetobacter baylyi
Proteus mirabilis

(b)

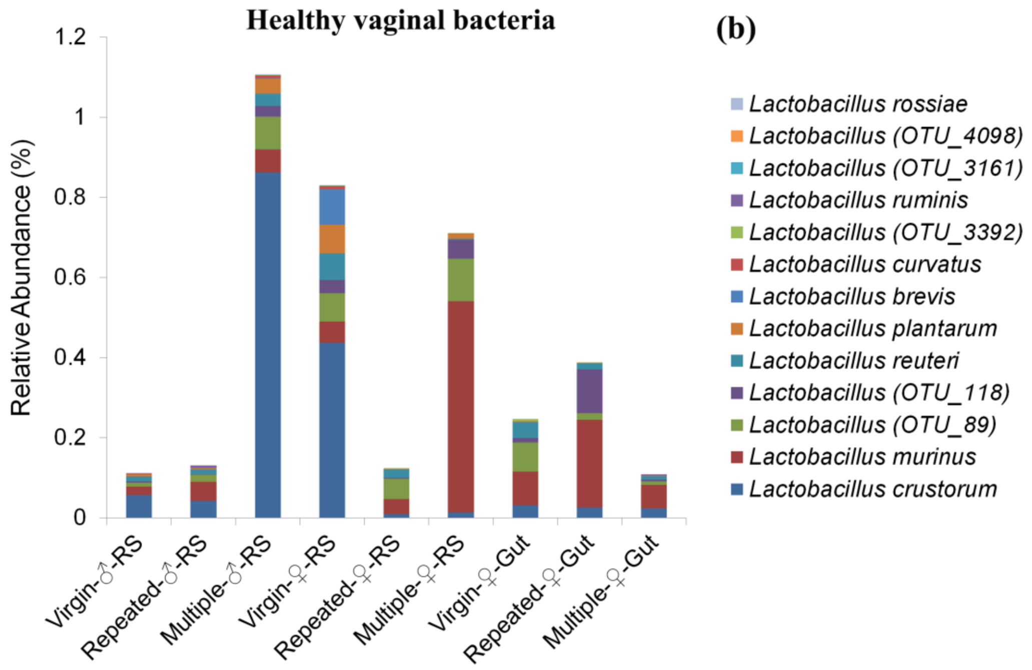
Healthy vaginal bacteria
Relative Abundance (%)
1.2
1
0.8
0.6
0.4
0.2
0
Virgin-♂-RS
Repeated-♂-RS
Multiple-♂-RS
Virgin-♀-RS
Repeated-♀-RS
Multiple-♀-RS
Virgin-♀-Gut
Repeated-♀-Gut
Multiple-♀-Gut
Lactobacillus rossiae
Lactobacillus (OTU_4098)
Lactobacillus (OTU_3161)
Lactobacillus ruminis
Lactobacillus (OTU_3392)
Lactobacillus curvatus
Lactobacillus brevis
Lactobacillus plantarum
Lactobacillus reuteri
Lactobacillus (OTU_118)
Lactobacillus (OTU_89)
Lactobacillus murinus
Lactobacillus crustorum

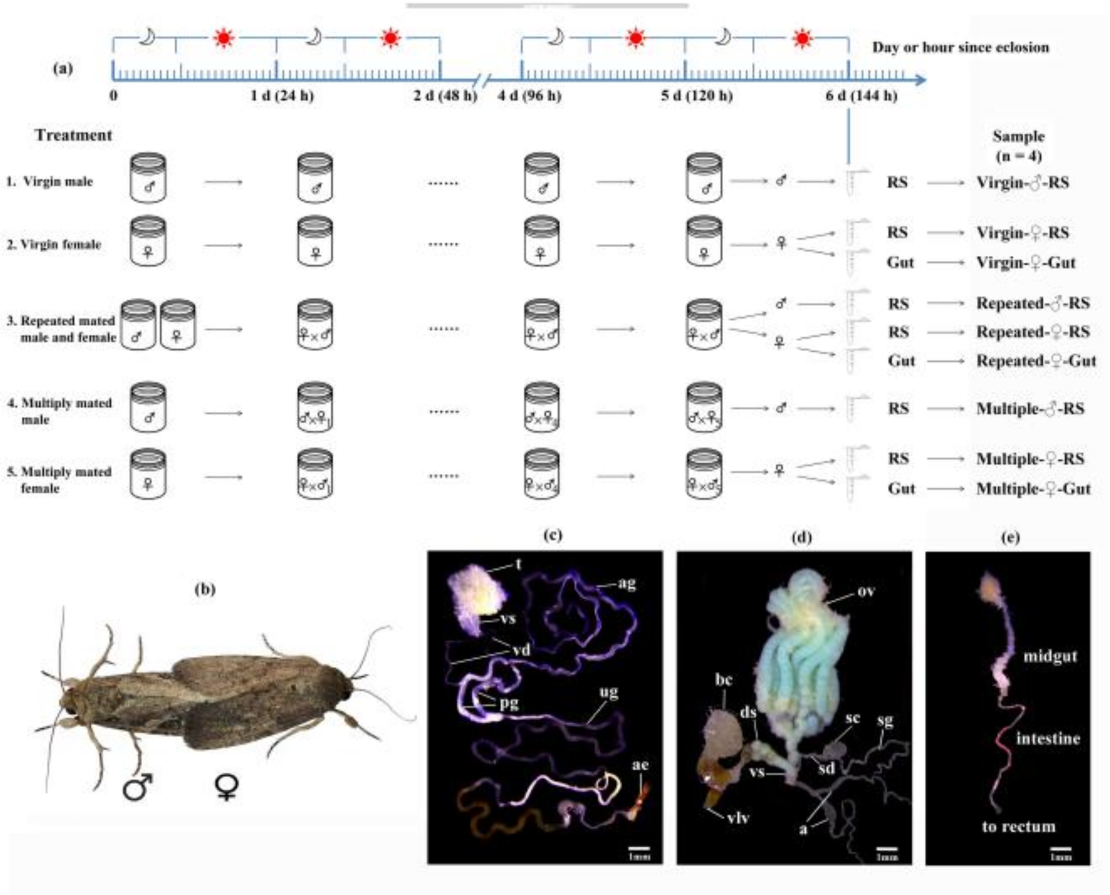
(a)
Day or hour since eclosion
0
1 d (24 h)
2 d (48 h)
4 d (96 h)
5 d (120 h)
6 d (144 h)
Treatment
Sample (n = 4)
1. Virgin male
2. Virgin female
3. Repeated mated male and female
4. Multiply mated male
5. Multiply mated female
RS
Gut
Virgin-♂-RS
Virgin-♀-RS
Virgin-♀-Gut
Repeated-♂-RS
Repeated-♀-RS
Repeated-♀-Gut
Multiple-♂-RS
Multiple-♀-RS
Multiple-♀-Gut
(b)
♂
♀
(c)
t
ag
vs
vd
pg
ug
ae
1mm
(d)
ov
bc
ds
sc
sg
vs
sd
vlv
a
1mm
(e)
midgut
intestine
to rectum
1mm

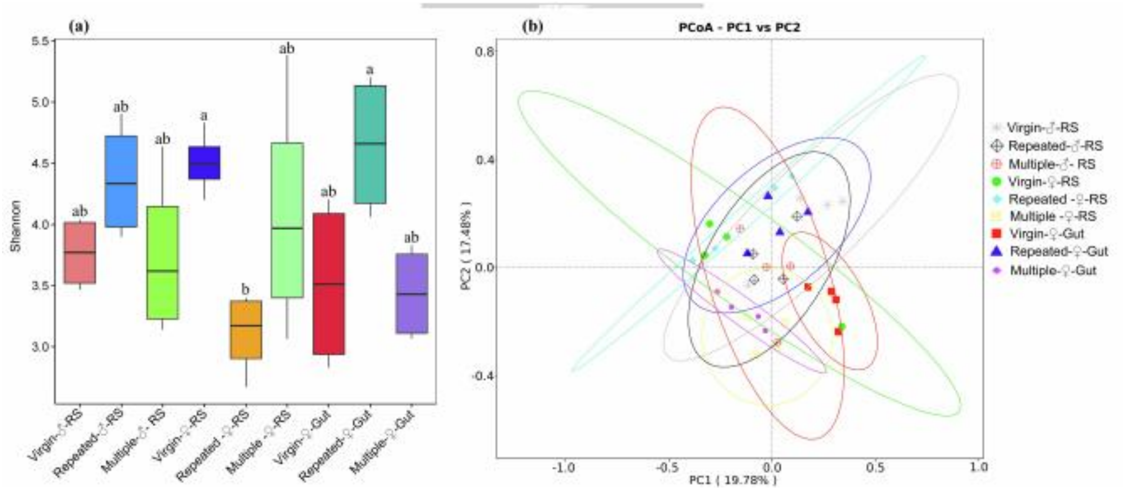
(a)
Shannon
5.5
5.0
4.5
4.0
3.5
3.0
ab
ab
ab
a
b
ab
ab
a
ab
Virgin-♂-RS
Repeated-♂-RS
Multiple-♂-RS
Virgin-♀-RS
Repeated-♀-RS
Multiple-♀-RS
Virgin-♀-Gut
Repeated-♀-Gut
Multiple-♀-Gut
(b)
PCoA - PC1 vs PC2
0.8
0.4
0.0
-0.4
PC2 (17.48%)
-1.0
-0.5
0.0
0.5
1.0
PC1 (19.78%)
Virgin-♂-RS
Repeated-♂-RS
Multiple-♂-RS
Virgin-♀-RS
Repeated-♀-RS
Multiple-♀-RS
Virgin-♀-Gut
Repeated-♀-Gut
Multiple-♀-Gut
